# Little Peacemakers: Microbes Can Promote Nonviolent Conflict Resolution by Their Hosts

**DOI:** 10.1002/ece3.71129

**Published:** 2025-04-16

**Authors:** Yonatan Bendett, Lilach Hadany

**Affiliations:** ^1^ School of Plant Sciences and Food Security Tel Aviv University Tel Aviv Israel

**Keywords:** aggressiveness, conflict resolution, Hawk‐Dove game, host‐microbes interactions, mathematical model, microbiome

## Abstract

Conflicts between individuals of the same species are common in nature and are mostly resolved with limited aggression. Several theoretical studies, such as the Hawk–Dove (HD) game model, investigate the evolution of limited aggression expressed during conflicts between individuals. These studies mainly focus on the individuals involved in the conflict and their genes. Recently accumulating evidence indicates that microbes are associated with diverse functions of their host and can affect host behavior. Here we extend the classic HD game model to include both the hosts and their microbes, examining how natural selection acts on the microbes. We find that nonaggressive host behavior is more likely to evolve and spread in a population when induced by the microbes residing in the host, compared to nonaggressive behavior induced by host genes. Horizontal transmission allows microbes to colonize new hosts, making their success dependent on the fitness of both the host and its opponent. Therefore, selection on the microbes favors reduced host aggressiveness under wider conditions compared to selection acting on genes alone. Our results suggest that microbes may help explain the ubiquity of nonviolent conflict resolution. Consequently, factors that alter the microbial composition within hosts may affect the aggressiveness level in host populations.

## Graphical abstract

Nonviolent conflict resolution is an open evolutionary question. Here we use mathematical modeling and investigate the possible role of microbes in conflict resolution between their hosts. We find that microbes may evolve to lower aggressiveness in conflicts, suggesting a new dimension to the question and to the modeling of host behavior.

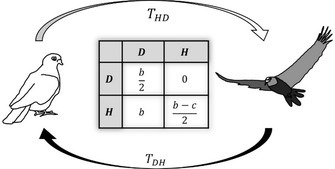

## 1 Introduction

Conflicts between individuals of the same species are common in nature (Abrams 2022; Hardy and Briffa 2013; Davies et al. 2012). The results of competition over food (Vogel 2005), territory (Festa‐Bianchet et al. 1990), mates (West‐Eberhard 1979), dominance rights (Chase 1974) and other goods may be critical for the survival of the individuals. However, these conflicts are often characterized by a “limited war” which restrains aggressiveness and reduces harm to involved individuals (Smith 1974; Treisman 1977). Three major theories were proposed to explain this phenomenon, including group selection, which claims that aggressive behavior may reduce the survival of the species (Huxley 1966); kin selection, proposing that aggressive individuals will likely harm their close relatives (Hamilton 1964; Smith 1964); selection at the individual level, where key factors are frequency‐dependent selection and repeated interactions between individuals (Smith and Price 1973; Smith 1974, 1982; Smith and Parker 1976); and combinations of the above theories (Grafen 1979; Hines and Smith 1979). Natural selection acting on the genes of the interacting individuals is at the heart of these theories. Here we suggest examining the effect of selection not only on the genes of the individuals in conflict but also on the microbes these individuals host.

Most organisms host abundant and diverse microorganisms (McFall‐Ngai et al. 2013; Rosenberg 2021). The involvement of microbes with the health and behavior of their hosts has been unraveled mainly in the last two decades (McFall‐Ngai et al. 2013; Li et al. 2020; Rosenberg 2021). Accumulating evidence shows that the gut microbiome can have substantial effects on the function of the nervous system through several mechanisms (e.g., gut–brain axis, vagus nerve [Foster and Neufeld 2013; Forsythe et al. 2014; Cryan et al. 2019; dos Claudino Santos et al. 2023]), and can alter social behavior, anxiety, and risk taking (Cryan and Dinan 2012; Mayer et al. 2014; Sampson and Mazmanian 2015; Schretter et al. 2018; Sharon et al. 2019; Bastiaanssen et al. 2020; Wu et al. 2021). The microbiome has been linked to aggression changes in fruit flies (Grinberg et al. 2022), dogs (Craddock et al. 2022), mice (Leclercq et al. 2017; Watanabe et al. 2021), and humans (Deng et al. 2022). Following microbial changes, reduced aggression was found in fruit flies and hamsters (Sylvia et al. 2017; Jia et al. 2021), and increased aggression was found in mice (Leclercq et al. 2017; Watanabe et al. 2021; Grinberg et al. 2022). Fecal microbiome transplants from antibiotic‐exposed infants increased aggression in mice compared to transplants from unexposed infants, suggesting a link between aggression and microbiome depletion (Uzan‐Yulzari et al. 2024). The host behavior can also affect the probability of the microbes' transmission to the offspring of their hosts, as well as to other hosts during social interactions (Kuthyar et al. 2019). Therefore, microbial manipulation of hosts' behavior in a way that improves the transmission of microbes (Poulin 2010; Dass et al. 2011; Archie and Tung 2015; Kuthyar et al. 2019) can be favored by natural selection on the microbes.

Studies indicate that shared environment as well as direct physical contact between social partners may play a key role in the transmission of microbial species (Archie and Tung 2015; Tung et al. 2015; Grieneisen et al. 2017; Dowd and Renson 2018; Sarkar et al. 2020). Behaviors like parental care and offspring grooming are known to increase microbial transmission from parents to offspring (Kulkarni and Heeb 2007; Chen et al. 2020; Kouete et al. 2023). A contest over a resource can be gradual, with no immediate aggressive actions. Often individuals display and can be engaged in a physical contact that does not settle the contest or cause serious harm. Examples are gentle scratching, bites, wrestling, and mutual pecking. These actions, along with escalated aggressive behavior, often involve exchange of body fluids, skin contact, or exposure to wounds, which can provide pathways for microbes to spread horizontally between individuals (Grice and Segre 2011; Kort et al. 2014; Archie and Tung 2015; Brito et al. 2019; Sarkar et al. 2020). Even when the interaction is characterized by ritualized displays without a direct contact, the involved individuals usually share their environment for a long time until the conflict is resolved (Smith 1974; Bishop and Cannings 1978), facilitating transmission through the shared environment.

We propose that natural selection on microbes may favor shaping their host aggressiveness while participating in conflicts, and that may help explain the dynamics of conflict resolution. In natural systems, both genes and microbes may affect host behavior. However, the genetic equilibrium resulting from selection acting solely on the genes is well studied (Smith and Price 1973; Smith 1974, 1982; Smith and Parker 1976). Here we investigate the effect of selection acting on the microbes, focusing on the case where aggressiveness is determined only by the microbes residing within the host. We expect the actual level of aggressiveness to lie between the two extreme cases, determined by a “tug of war” between genes and microbes. While host genes are transmitted only during reproduction and only through vertical transmission, from parent to offspring, microbes can also be transmitted throughout the host life horizontally, from one host to another during host interactions (Duranti et al. 2017; Shade et al. 2017; Moeller et al. 2018; Robinson et al. 2019; Valles‐Colomer et al. 2022; Liu et al. 2023). Therefore, it seems that for the microbes, social interaction of their host entails two aspects: the nature of the interaction itself, with its effect on host fitness (affecting microbes' vertical transmission); as well as the consequences of horizontal transmission during interaction—the opportunity to be transmitted to another host. In that case, microbes' vertical transmission would be affected by the fitness of both interacting hosts. We use population genetics and game‐theory models to investigate this hypothesis, and find that microbes are likely to evolve to induce nonviolent resolution of conflicts by the host.

## 2 Model and Results

### 2.1 Model 1: Microbe Dependent Hawk–Dove (mHD) Game With Pure Strategies

**FIGURE 1:**
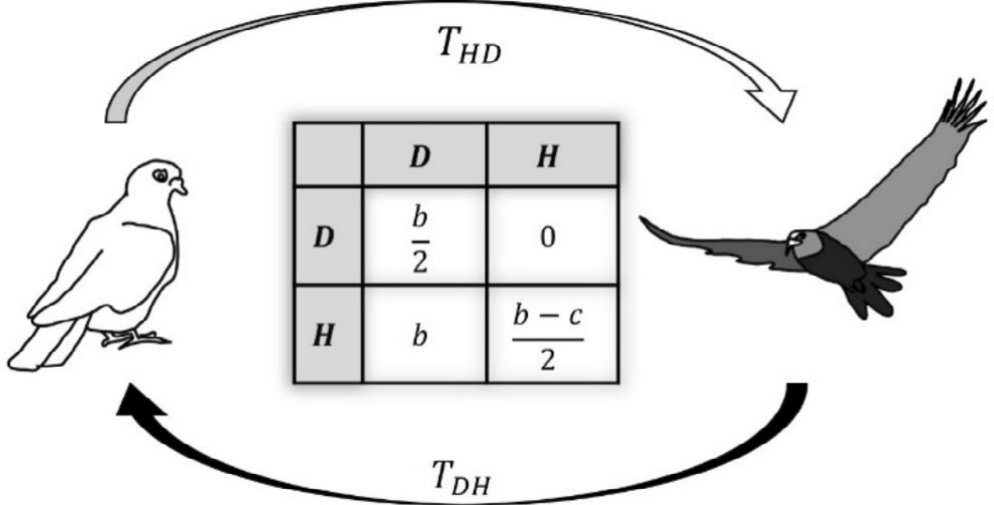
Payoff matrix and illustration of microbes‐dependent Hawk–Dove (mHD) game. Individuals interact randomly in pairs with a Hawk–Dove (HD) game payoff. During host interaction, microbes can be transmitted between the interacting hosts. The fitness benefit to the host from obtaining a resource is denoted by b, the fitness cost due to injury during aggressive interaction is denoted by c, and $T_{\mathrm{ij}}$ stand for the probability of transmission and establishment of microbe i during its host interaction with a host carrying microbe of type j.

We consider an infinite, fully mixed population of asexual individuals. Individuals interact randomly in pairs, with a Hawk–Dove (HD) game payoff (Figure 1), where each individual participates in one interaction every generation. We assume that all individuals share a genetic background and a standard composition of microbes determining a baseline level of aggressive behavior. On top of that, each individual in the population can carry one of the following two microbes: Microbes of type H induce their host to behave aggressively, that is, behave always like a “Hawk.” Microbes of type D induce their host to behave in a nonaggressive way, that is, behave always like a “Dove.” During host interaction, microbes can be transmitted between the interacting hosts. Transmission probabilities for the different microbe types are denoted by $T_{\mathrm{HD}}$ and $T_{\mathrm{DH}}$ (assumed to be less than 0.5). $T_{\mathrm{HD}}$ represents the probability of microbes of type H being transmitted to a host carrying microbe of type D, taking over its niche, and likewise for $T_{\mathrm{DH}}$. When two hosts carrying the same microbe type interact, horizontal transmission effectively does not alter their microbial composition. At the end of each generation, individuals reproduce according to their fitness. Microbes are transmitted vertically from parent to offspring and mutations are neglected. Finally, the offspring generation replaces the parent generation.

We first compare the evolution of microbe‐induced aggression with the classic case of genetically encoded aggression in the host.

The outcomes of the genetic HD game are well known (Smith and Price 1973; Smith 1982). A gene for Hawk‐like behavior takes over the population when the benefit from obtaining the resource is greater than the cost of being injured (b>c). In the opposite case, the population reaches a stable polymorphism, where the proportion of Hawk‐gene is b/c and the proportion of Dove‐gene is 1−b/c.

Analyzing the special case of aggression affected by the microbes with symmetric horizontal transmission ($T_{\mathrm{HD}}=T_{\mathrm{DH}}=T$), we find that the equilibrium states and their stability conditions depend on the horizontal transmission probability T (see Sections 4.1, 4.2). Microbe D can reach a stable polymorphism with microbe H when:

(1.1) $$\;T>\frac{b-c}{2b}$$

The proportion of microbe D in the polymorphic state is:

(1.2) $$\;{\hat{X}}_{D}=1-\frac{b}{c}\;\left(1-2T\;\right)$$

**FIGURE 2:**
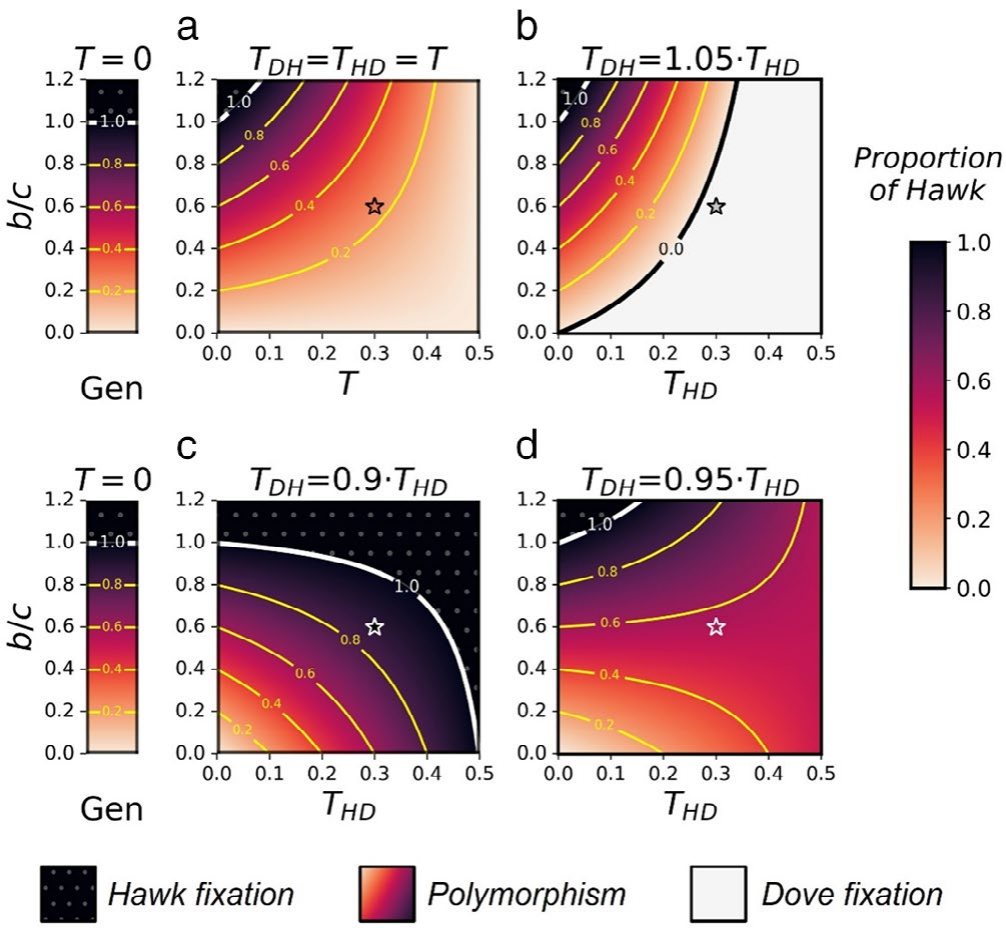
Horizontal transmission rates affect the stable equilibria of the Hawk–Dove game. The expected equilibrium proportion of hosts carrying microbe H in population, for different b/c ratio (*y*‐axis) and different values of horizontal transmission probability (*x*‐axis), plotted for (a) $T_{\mathrm{DH}}/T_{\mathrm{HD}}=1$, (b) $T_{\mathrm{DH}}/T_{\mathrm{HD}}=1.05$, (c) $T_{\mathrm{DH}}/T_{\mathrm{HD}}=0.9$, and (d) $T_{\mathrm{DH}}/T_{\mathrm{HD}}=0.95$. c=0.1. The black dotted area represents the parameter range where microbe H takes over the population, the colored area represents the parameter range where microbe H and microbe D reach stable polymorphism, and the white area represents the parameter range where microbe D takes over the population. Note that (a) presents the symmetrical transmission case, (b) and (d) biased transmission to similar levels at different directions. The stars refer to the dynamics shown in Figure 3. For derivation see Section 4.1, 4.2.

With T>0, the equilibrium proportion of microbe D in the population increases compared to the case of T=0 (and the genetic case), (Figure 2a), and nonaggressive behavior (“Dove”) increases with it. The population converges to the same equilibrium from any positive frequencies of microbes D and H. Intuition for the result can be obtained from the following argument: horizontal transmission is influential where a host carrying microbe H interacts with a host carrying microbe D. In the genetic case, a HD interaction is profitable for the “Hawk” and not to the “Dove.” From the microbe's perspective, horizontal transmission results in taking the niche of a host that previously carried another microbe type, potentially gaining from the host's benefit from the HD interaction. Since horizontal transmission is symmetric, hosts exchange occurs at the same rate in both directions, and on average it results in a greater relative profit for microbe D.

Note that when T=0, meaning no horizontal transmission, condition (1.1) reduces to b<c corresponding to the genetic HD game model.

### 2.2 Pure Strategies Model With Unequal Horizontal Transmission

Next, we consider the case where horizontal transmission is not symmetric and account for different transmission probabilities for the different microbe types, denoted by $T_{\mathrm{HD}}$ and $T_{\mathrm{DH}}$ (Figure 1).

We find that the parameter range can be divided to three areas (see Sections 4.1, 4.2). Any advantage of microbe D in horizontal transmission ($T_{\mathrm{DH}}>T_{\mathrm{HD}}$) increases the proportion of Dove‐like behavior at equilibrium, compared to the genetic case, which can even reach a stable fixation in the population (Figure 2b). This result is contrary to the genetic case where a gene for Dove‐like behavior cannot fixate.

When $T_{\mathrm{HD}}>T_{\mathrm{DH}}$ the result is more complex. When microbes of type H exhibit a sufficiently large advantage in horizontal transmission (see Section 4.2) this advantage can outbalance the relative profit for microbes of type D from successful transmissions, and the equilibrium frequency of microbe H can be higher than in the genetic case (Figure 2c). When the advantage is not large enough (see Section 4.2), an intermediate state can be observed (Figure 2d).

### 2.3 Pure Strategies Model With Imperfect Vertical Transmission

So far, we assumed that the vertical transmission of microbes is perfect, so the offspring receives its parent's microbe with probability 1. Here, we generalize the model accounting for imperfect vertical transmission: With probability VT an offspring inherits its parent's microbe, and with probability 1−VT it acquires a random microbe from the parent population.

**FIGURE 3:**
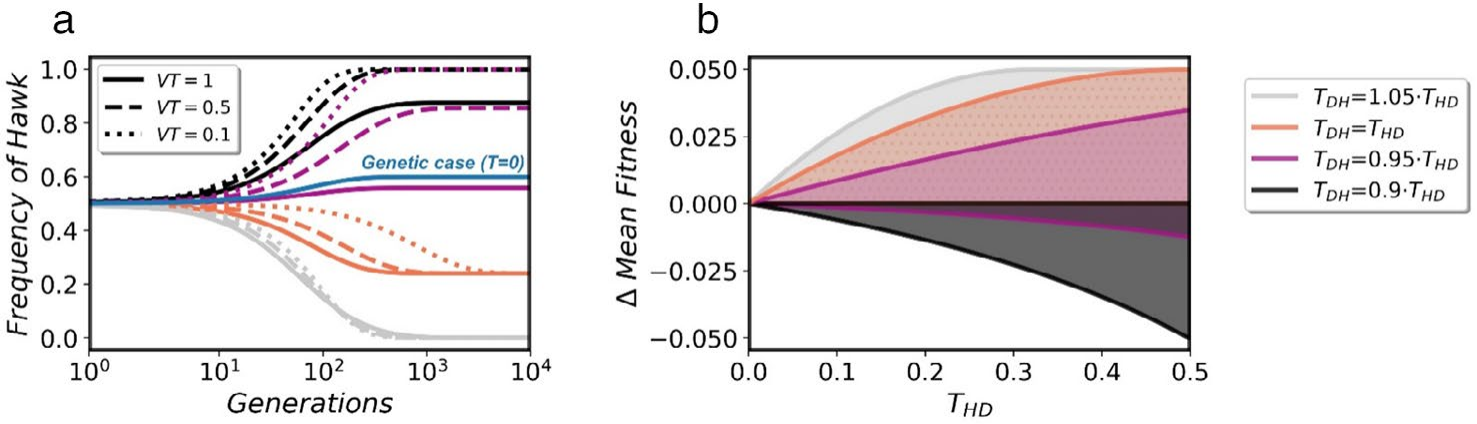
Vertical transmission and mean fitness in the mHD game model. (a) The estimated frequency of microbe H in the population as function of generations number, plotted for different values of vertical transmission probability of the microbes (VT). Solid lines represent perfect vertical transmission VT=1, dashed lines VT=0.5, and the dotted lines VT=0.1. The blue line refers to the genetic case ($T_{\mathrm{HD}}=T_{\mathrm{DH}}=0$, VT=1), and the colors of the other lines correspond to the stars shown in Figure 2, with different ratios of $T_{\mathrm{DH}}$ and $T_{\mathrm{HD}}$, and $c=0.1,b=0.06,T_{\mathrm{HD}}=0.3$. See Sections 4.1, 4.3. (b) The mean fitness differences between the mHD game and the genetic HD game (y‐axis) evaluated in the stable equilibrium state ($\Delta \bar{\omega }={\bar{\omega }}_{mHD\;game}-{\bar{\omega }}_{\text{genetic}\;HD\;game}$), as function of horizontal transmission probability of microbe H (*x*‐axis), plotted for c=0.1, and different $T_{\mathrm{DH}}/T_{\mathrm{HD}}$ ratios. The black area represents the mean fitness differences range for different b values and for $T_{\mathrm{DH}}/T_{\mathrm{HD}}=1$, the reddish area $T_{\mathrm{DH}}/T_{\mathrm{HD}}=0.95$, the orange $T_{\mathrm{DH}}/T_{\mathrm{HD}}=1$, and the gray $T_{\mathrm{DH}}/T_{\mathrm{HD}}=1.05$.

We find that vertical transmission of microbes may alter the equilibrium states and their stability, as well as the rate of convergence (Figure 3a). In general, the next generation frequencies are affected by two aspects of the hosts interactions: the benefits and the costs to the hosts following the interactions, as well as the asymmetric horizontal transmission of the microbes. The higher the vertical transmission probability, the more influential the change in hosts fitness following the interactions, compared with asymmetric microbial transmission, and vice versa. In the special case of symmetric horizontal transmission, vertical transmission of microbes does not alter the equilibrium states and their stability, but it does affect the rate of convergence: the higher the vertical transmission probability, the faster the rate of convergence to the equilibria. A general model of mHD game with imperfect vertical transmission is included in Sections 4.1, 4.3.

### 2.4 Mean Fitness in the Pure Strategies Model

In any version of the HD model, mean fitness increases with the frequency of Dove (see Equation (4)). Thus, in our model, whenever mHD leads to increased frequency of D (Figure 2), it further leads to increased mean fitness (Figure 3b). Differently from the genetic case, mHD may even result in maximal fitness for the game with stability of Dove fixation (Figure 3b). However, when mHD results in higher Hawk frequency compared to the genetic case, it leads to lower mean fitness (Figures 2c and 3b black area). In that case, the hosts experience more damage from injuries, against the genes' best interests.

### 2.5 Model 2: Mixed Strategies Model

So far, we have assumed that each gene or microbe induces one pure strategy: Hawk‐like behavior or Dove‐like behavior. However, individuals may also be able to alternate between aggressive and nonaggressive behavior, or to adopt an intermediate level of aggressiveness. A mixed strategy can thus be seen as a developmental strategy determined once (either of the two extreme behaviors), or as an intermediate behavior between them (with the mean probability that characterizes the strategy). Here we examine the case where individuals are able to adopt mixed strategies, determined either genetically or microbially.

In the classical genetic model, selection favors a gene that induces a mixed strategy with frequencies corresponding to the equilibrium proportions of the pure strategies presented earlier, b/c (see Section 4.4), whenever the risk of injury during interaction is relatively high (c>b). Here we investigate the evolvability of a microbe that induces its host to adopt a different mixed strategy on that background.

Let A and B be microbes that determine host mixed strategies as follows: microbe A induces its host to play “Hawk” with probability $P_{A}\left(H\right)$ and to play “Dove” with probability $P_{A}\left(D\right)=1-P_{A}\left(H\right)$. Microbe B is assumed to have no effect on the host strategy, hence its host adopts the stable genetic mixed strategy: play “Hawk” with probability b/c and play “Dove” with probability 1−b/c. We assume that each host carries one microbe type: A or B. Hosts interact randomly in pairs with a HD game payoff, microbes can be transmitted between the interacting hosts with equal probabilities ($T_{A}=T_{B}=T$), and vertical transmission of the microbes is perfect (VT=1).

**FIGURE 4:**
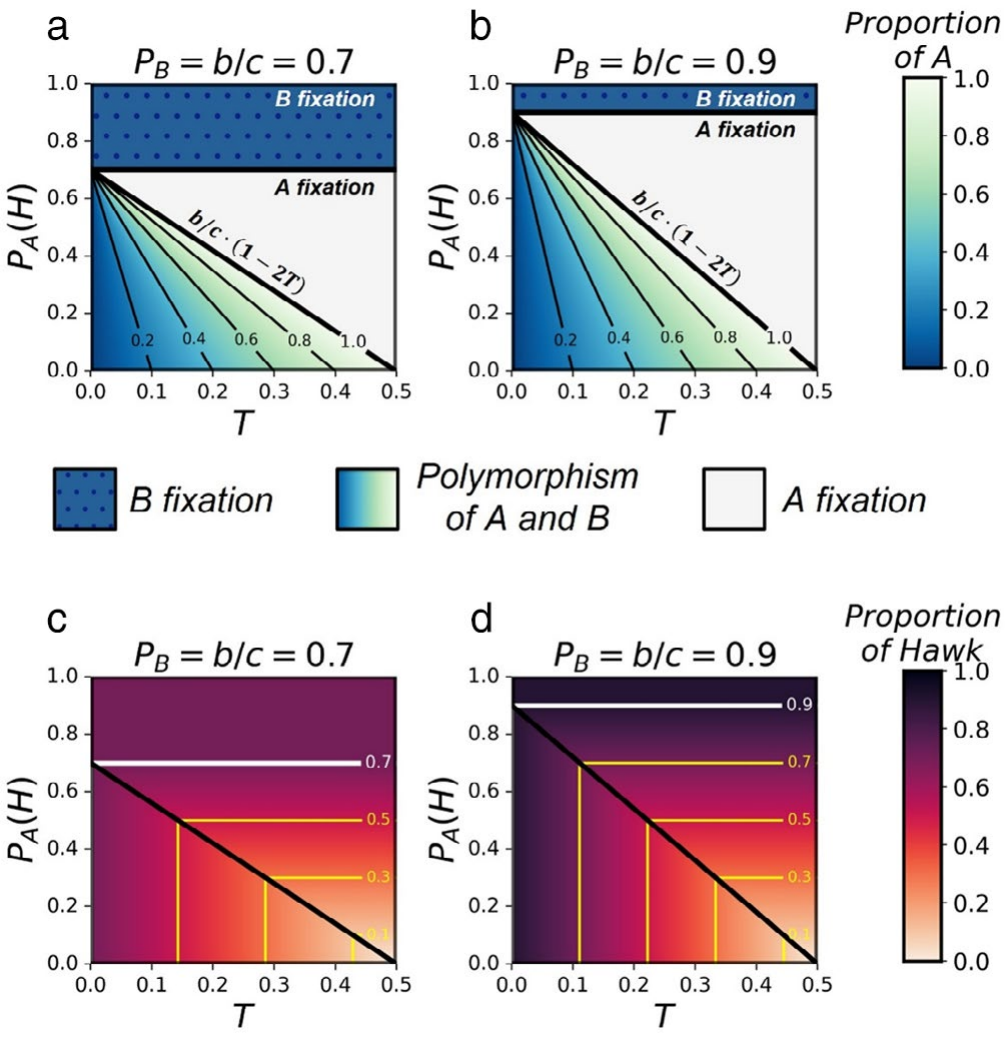
Equilibrium stable states of the population when there is a competition between the genes mixed strategy and an alternative mixed strategy induced by a microbe. (a and b) The expected proportion of hosts carrying microbe A in population, based on equilibrium and stability analysis of the mixed strategies model of microbes Hawk–Dove game, for different Hawk‐strategy frequencies induced by microbe A (*y*‐axis) and different values of horizontal transmission probability (*x*‐axis), plotted for (a) $P_{B}\left(H\right)=b/c=0.7$ and (b) $P_{B}\left(H\right)=b/c=0.9$. The blue dotted area represents the range of parameters in which microbe A cannot evolve, the colored area represents a range of parameters in which microbe A and microbe B reach stable polymorphism, and the white area represents the range of parameters in which microbe A takes over the population. The aggression threshold $\frac{b}{c}\;\left(1-2T\right)$ is shown as well. When $P_{A}\left(H\right)<P_{B}\left(H\right)=\frac{b}{c}$, the threshold determines whether microbe A fixates in the population ($P_{B}\left(H\right)>P_{A}\left(H\right)>\frac{b}{c}\;\left(1-2T\right)$) or reaches stable polymorphism with microbe B ($P_{A}\left(H\right)<\frac{b}{c}\;\left(1-2T\right)<P_{B}\left(H\right)$). For derivation see Sections 4.5, 4.6. (c and d) The expected proportion of Hawk‐like behavior in the population in the equilibrium stable state. (c) Corresponds to (a), and (d) corresponds to (b), plotted for $P_{B}\left(H\right)=b/c=0.7$ and $P_{B}\left(H\right)=b/c=0.9$, respectively.

We find (see Sections 4.5, 4.6) that when microbe A induces less aggressive behavior than the genes ($P_{A}\left(H\right)<P_{B}\left(H\right)$), it can evolve and either reach stable polymorphism with microbe B or fixate in the population. Within this parameter range, there is a level of aggression threshold $\frac{b}{c}\left(1-2T\right)$, that corresponds to the level of aggression of the stable mixed strategy in the mHD game model (see Section 4.4). When microbe A induces higher aggression level then the threshold (but lower than microbe B), microbe A can take over the population, while below this threshold it reaches polymorphism with microbe B. As horizontal transmission probability increases, microbe A can fixate even while inducing less aggressive behavior, and reach a higher expected proportion in the stable polymorphic state (Figure 4a,b). As a result, the total proportion of aggressive behavior in the population, exhibited either by hosts carrying microbe A or hosts carrying microbe B, decreases as the horizontal transmission probability increases (Figure 4c,d). However, when microbe A induces more aggressive behavior than the genes ($P_{A}\left(H\right)>P_{B}\left(H\right)$), it cannot invade a population carrying microbe B, for any horizontal transmission probability. Therefore, the maximum proportion of aggressive behavior in the population is determined by the genetic strategy induced by microbe B. Nevertheless, if microbe A has a sufficiently large advantage in horizontal transmission ($T_{A}>T_{B}$), we expect microbe A to evolve even when inducing more aggressiveness compared to microbe B.

## 3 Discussion

In this work, we present a new perspective on the evolution of limited aggression during conflict resolution. Our models suggest that microbes can play a role in promoting nonaggressive behavior in their hosts. Our results predict that introducing symmetric horizontal transmission of the microbes facilitates the evolution of limited aggression during the resolution of conflicts (Figure 2a). Compared to the case of gene‐encoded aggression, aggressive behavior is expected in lower proportions when aggressive behavior is induced by microbes (Figures 2a and 3b), accompanied by increased mean fitness of the population. These results are obtained whether microbes induce pure strategies or mixed ones. Our models extend the classic HD game model, which is one of the theoretical models dealing with the rarity of excessive aggression during conflicts. The HD game model shows that aggression is reduced when the aggressive strategy (“Hawk”) carries a heavier cost of injury than the potential benefit.

The behavior of hosts can be affected by many factors, including the host's genes, as well as the microbes residing in the host. The classic HD game model considers an extreme case where aggressive behavior is determined only by the individuals' genes. Here we focus on the other extreme: we consider a case where individuals are similar in all respects other than the type of microbe they carry. We focus on the effect of these microbes on the aggressiveness level of their hosts, investigating how natural selection acts on these microbes. We expect that in the case of microbe‐induced aggression, not only the fitness of the current host of the microbe should be maximized (as in the genes case), but the fitness of the contestant and the transmission probabilities should be taken into account as well. That is since microbes demonstrate both vertical and horizontal transmission. When passing a microbe from parent to offspring through vertical transmission, the fitness of a parent determines the maximum number of offspring that the microbe can reside in. Therefore, when the only mode of transmission is vertical (as in the genetic case), the success of the inherited elements (genes or microbes) depends on the reproductive success of the parent. When introducing also horizontal transmission during an interaction of hosts, there is a probability of being transmitted to the other host, as well as a probability of losing the niche in the current host. Therefore, the success of a microbe depends on the fitness of the two interacting hosts. From the microbe's perspective, the reproductive success of each host should be optimal, according to the probability of inhabiting it after the interaction. We show that these two modes of transmission lead to selection in favor of microbes that induce less aggression under wider conditions, compared to the genetic case.

Our work can be extended in several ways. First, we compared here two different scenarios: genes‐encoded and microbes‐encoded aggression. However, it is reasonable to assume that both selection levels affect the evolution of aggressive behavior, leading to behavior in between the stable states of the extreme cases. The selective forces acting on the hosts genes and the microbes may also act in opposite directions (e.g., one pulls toward increasing aggressiveness and the other toward reducing aggressiveness). This tug of war can be decided in different ways, depending on the strength of selection pressures (van Vliet and Doebeli 2019; Kolodny et al. 2020). In addition, an arms race between genes and microbes may evolve, and previous theoretical models considered the possibility of genes developing resistance to microbes' manipulation (Lewin‐Epstein and Hadany 2020). Second, we focus on a simplified case, where changes in aggressiveness levels are determined by a single microbe type the hosts carry (D or H, A or B). However, the behavior of hosts can be affected by the entire microbiota, where different microbes can impact aggressiveness to varying degrees and in different directions, with distinct transmission probabilities. The microbial composition within the hosts can be modeled in more realistic way, by accounting for the various microbes' types that inhabit the microbiota, their characteristics, the relationships between them and their effects on the host (Zeng et al. 2017; Lewin‐Epstein et al. 2023; Roughgarden 2023). Furthermore, the influence of microbes on host behavior can be modeled in alternative ways. We assumed that microbes affect the behavioral strategy (Hawk or Dove), but their impact on the tendency to participate in more interactions could also be considered (Lavy et al. 2022). Third, we assume that all individuals share the same baseline condition and fitness, apart from the microbes they carry. However, the feasibility of a strategy can be condition dependent. Therefore, incorporating external and internal factors, like age and stress, may reveal additional insights. Fourth, by incorporating horizontal transmission, mHD game models take into account the mutual fitness effects of the competing hosts, similarly to HD game between relatives (Grafen 1979; Hines and Smith 1979). We assume that there is no genetic relatedness between hosts. However, accounting for both genetic relatedness and “microbial relatedness” may reveal a linkage between them, since it is empirically known that relatives often share similar microbes (Turnbaugh et al. 2009; Yatsunenko et al. 2012).

Our work joins several theoretical and empirical studies that investigated the involvement of microbes in their host functioning. Several theoretical studies explored the potential role of microbes in shaping their hosts social characteristics, including cooperation (Lewin‐Epstein and Hadany 2020; Lewin‐Epstein et al. 2017; van Vliet and Doebeli 2019) and resource sharing (Rog et al. 2022), paternal care (Gurevich et al. 2020), aggregation of insects (Lavy et al. 2022) and addictive behavior (Lewin‐Epstein et al. 2023). In addition, accumulating empirical studies reveal the impact of microbes on diverse functions of hosts and the underlying biological mechanisms.

The foundations of our model can be applied to other systems with both horizontal and vertical transmission, such as culture (Danchin et al. 2011; Jablonka and Lamb 2014; Bonduriansky and Day 2020). Culture, like microbes, can be inherited from parents, as well as acquired during interactions with other individuals (Cavalli‐Sforza and Feldman 1981; Rushton et al. 1986; Kolodny et al. 2018; Cohen et al. 2021; Denton et al. 2023; Borofsky et al. 2024). Our results suggest that a culture that encourages nonaggressive behavior can spread in populations, especially if there is a strong link between transmissions and interactions (e.g., by education, learning, imitation, so forth).

Our theoretical results, that nonaggressive conflict resolution may be regulated by the microbes that the individuals host, call for further empirical examination: exploration of whether altering the microbial composition (by using antibiotics, transplantation, etc.) results in behavioral changes in the context of conflict resolution, and what mechanisms are involved. Our findings further suggest that the rate of microbial transmission, both horizontal and vertical transmission, could affect the level of aggression expressed during conflicts. We therefore predict that populations with different social structures (food sharing, parental care, co‐sheltering) may evolve to express different levels of aggressiveness during conflicts.

This study highlights the potential role of microbes in shaping conflict resolution between their hosts and suggests that microbes may help explain the widespread occurrence of nonviolent conflict resolution. It demonstrates how selection on the microbes may favor manipulation of host behavior, often resulting in a conflict of interest between host genes and their microbes. Hence, factors that modify the microbial composition within hosts may significantly shape the aggressiveness levels observed in host populations.

## 4 Methods

### 4.1 The General Model of the Pure Strategies mHD Game

In this section, we describe the complete model that is presented in Figures 1 and 2 in the main text. Here we assume, that each type of microbe has a different horizontal transmission probability ($T_{\mathrm{DH}},T_{\mathrm{HD}}$), and that vertical transmission (VT) can be imperfect (Table 1). We show here the derivations with population genetics approach. Parallel analysis using evolutionary game theory yields similar results (see Section 4.4).

**TABLE 1:** Parameters of mHD game model.

Parameter	Description
$X_{H}$	Proportion of hosts carrying a standard microbiome plus microbes of type H
$X_{D}$	Proportion of hosts carrying a standard microbiome plus microbes of type D
b	Fitness benefit to the host following obtaining a resource
c	Fitness cost to the host due to injury during an interaction
$T_{\mathrm{HD}}$	Transmission and establishment probability of microbe H during its host interaction with a host carrying microbe of type D
$T_{\mathrm{DH}}$	Transmission and establishment probability of microbe D during its host interaction with a host carrying microbe of type H
VT	Vertical transmission probability of a microbe from parent to offspring during reproduction

*Note:* The parameters of the general mHD model that is presented in the main text in Figures 1, 2, 3.

We assume that interactions occur randomly in pairs. When two individuals with Hawk‐like behavior interact, each contestant has a 50% chance of injuring its opponent and obtaining the resource (b), and a 50% chance of being injured. When two individuals with Dove‐like behavior interact, the resource is shared equally by the contestants (alternatively, the chance to obtain the resource is 50%). When individuals with different behavioral strategies interact, the “Hawk” obtains the resource, and the “Dove” retreats before being injured.

During host interaction, microbes can be transmitted between the interacting hosts with probabilities $T_{\mathrm{HD}}$ and $T_{\mathrm{DH}}$. $T_{\mathrm{HD}}$ represents the probability of microbes of type H being transmitted to a host carrying microbe of type D, taking over its niche, and likewise for $T_{\mathrm{DH}}$. At the end of each generation, individuals reproduce according to their fitness. Microbes are transmitted vertically, and in general this vertical transmission (VT) can be imperfect. With probability VT an offspring inherits its parent's microbe, and with probability 1−VT it inherits a random microbe from the parent population. Then the offspring generation replaces the parent generation.

Using the above, the proportions of newborn hosts that produced by parents carrying microbe H and by microbe D are denoted by $\tilde{X_{H}}$ and $\tilde{X_{D}}$, respectively:

(2.1) $$\;\tilde{X_{H}}=\frac{X_{H}^{2}W\left(H,H\right)+X_{H}X_{D}W\left(H,D\right)}{\bar{\omega }}$$

(2.2) $$\;\tilde{X_{D}}=\frac{X_{D}^{2}W\left(D,D\right)+X_{D}X_{H}W\left(D,H\right)}{\bar{\omega }}$$

Where the fitnesses of the hosts following each interaction are:

(3.1) $$\;W\left(H,H\right)=1+0.5b-0.5c$$

(3.2) $$\;W\left(H,D\right)=\left(1+b\right)\left(1-T_{\mathrm{DH}}\right)+1\cdot T_{\mathrm{HD}}$$

(3.3) $$\;W\left(D,D\right)=1+0.5b$$

(3.4) $$\;W\left(D,H\right)=1\cdot \left(1-T_{\mathrm{HD}}\right)+\left(1+b\right)T_{\mathrm{DH}}$$

The mean fitness of the population in each generation is:

(4) $$\;\bar{\omega }=\sum_{i=H,D}X_{i}\sum_{j=H,D}X_{j}\cdot W\left(i,j\right)=1+\frac{b}{2}-X_{H}^{2}\frac{c}{2}$$

Thus, the proportions of newborn hosts carrying microbe H and microbe D in the next generation are:

(5.1) $$\;X_{H}^{\prime }=\tilde{X_{H}}\mathrm{VT}+\left(1-\mathrm{VT}\right)\left[X_{H}^{2}+X_{H}X_{D}\left(1+T_{\mathrm{HD}}-T_{\mathrm{DH}}\right)\right]$$

(5.2) $$\;X_{D}^{\prime }=\tilde{X_{D}}\mathrm{VT}+\left(1-\mathrm{VT}\right)\left[X_{D}^{2}+X_{H}X_{D}\left(1-T_{\mathrm{HD}}+T_{\mathrm{DH}}\right)\right]$$

where $X_{H}^{2}+X_{H}X_{D}\left(1+T_{\mathrm{HD}}-T_{\mathrm{DH}}\right)$ and $X_{D}^{2}+X_{H}X_{D}\left(1-T_{\mathrm{HD}}+T_{\mathrm{DH}}\right)$ are the proportions of hosts carrying microbes of type H, D, respectively, after interactions and before reproduction.

### 4.2 Equilibria and Stability of the Pure Strategies mHD Game With Perfect Vertical Transmission (VT=1)

The equilibrium states are the frequencies for which $X_{H}^{\prime }=X_{H}$ and $X_{D}^{\prime }=X_{D}$, or alternatively: $\Delta X_{H}=X_{H}^{\prime }-X_{H}=0$, and $\Delta X_{D}=X_{D}^{\prime }-X_{D}=0$. When $\Delta X_{H}=0$ is satisfied, $\Delta X_{D}=0$ is satisfied as well since $X_{D}=1-X_{H}$. This happens when:

(6) $$\begin{array}{cc} \;\Delta X_{H} & =X_{H}\frac{X_{H}W\left(H,H\right)+X_{D}W\left(H,D\right)-\bar{\omega }}{\bar{\omega }}\\ & =X_{H}\;X_{D}\frac{\left[\frac{b}{c}\;\left(1-2T_{\mathrm{DH}}\;\right)+\frac{2}{c}\;\left(T_{\mathrm{HD}}-T_{\mathrm{DH}}\;\right)\right]-X_{H}}{\frac{2}{c}\bar{\omega }}=0 \end{array}$$

Therefore, the equilibrium states are: ${\hat{X_{H}}}_{1}=1$, fixation of microbe H; ${\hat{X_{H}}}_{2}=0$, fixation of microbe D; and ${\hat{X_{H}}}_{3}=\frac{b}{c}\cdot \left(1-2T_{\mathrm{DH}}\;\right)+\frac{2}{c}\cdot \left(T_{\mathrm{HD}}-T_{\mathrm{DH}}\right)$, polymorphism of H and D. If horizontal transmission is equal ($T_{\mathrm{HD}}=T_{\mathrm{DH}}=T$), the polymorphic equilibrium gets the form of ${\hat{X_{H}}}_{3}=\frac{b}{c}\cdot \left(1-2T\right)$, and when there is no horizontal transmission ($T_{\mathrm{HD}}=T_{\mathrm{DH}}=0$) ${\hat{X_{H}}}_{3}=\frac{b}{c}$, which corresponds to the classic genetic case.

${\hat{X_{H}}}_{1}=1$ is stable when $\frac{2T_{\mathrm{HD}}+b-c}{2T_{\mathrm{HD}}\left(1+b\right)}>\frac{T_{\mathrm{DH}}}{T_{\mathrm{HD}}}$ ($\frac{b-c}{2b}>T$); ${\hat{X_{H}}}_{1}=0$ is stable when $\frac{2T_{\mathrm{HD}}+b}{2T_{\mathrm{HD}}\left(1+b\right)}<\frac{T_{\mathrm{DH}}}{T_{\mathrm{HD}}}$ (unstable); and ${\hat{X_{H}}}_{3}=\frac{b}{c}\left(1-2T_{\mathrm{DH}}\right)+\frac{2}{c}\left(T_{\mathrm{HD}}-T_{\mathrm{DH}}\right)$ is stable when $\frac{2T_{\mathrm{HD}}+b-c}{2T_{\mathrm{HD}}\left(1+b\right)}<\frac{T_{\mathrm{DH}}}{T_{\mathrm{HD}}}<\frac{2T_{\mathrm{HD}}+b}{2T_{\mathrm{HD}}\left(1+b\right)}$ ($\frac{b-c}{2b}<T<\frac{1}{2}$). The conditions for the case of $T_{\mathrm{HD}}=T_{\mathrm{DH}}=T$ are given in the parentheses (see Appendix S1: Note 1, 1.1, for stability analysis).

### 4.3 Pure Strategies mHD Game With Imperfect Vertical Transmission

When both types of microbes are not perfectly transmitted to the next generation (VT<1), by using Equations (2.1), (2.2), (3.1), (3.2), (3.3), (3.4), (4), (5.1), and (5.2), $X_{H}^{\prime }$ gets the form of:

(7) $$\begin{array}{cc} \;X_{H}^{\prime }= & \mathrm{VT}\frac{X_{H}^{2}\left(1+\frac{b}{2}-\frac{c}{2}\right)+X_{H}X_{D}\left(\left(1+b\right)\left(1-T_{\mathrm{DH}}\right)+T_{\mathrm{HD}}\right)}{1+\frac{b}{2}-X_{H}^{2}\frac{c}{2}}\\ & +\left(1-\mathrm{VT}\right)\left[X_{H}^{2}+X_{H}X_{D}\left(1+T_{\mathrm{HD}}-T_{\mathrm{DH}}\right)\right] \end{array}$$

The equilibrium states are the frequencies that satisfy $\Delta X_{H}=X_{H}^{\prime }-X_{H}=0$. This happens when:

(8) $$\;\Delta X_{H}=\frac{X_{H}X_{D}\left[X_{H}^{2}\left(1-\mathrm{VT}\right)\left(T_{\mathrm{DH}}-T_{\mathrm{HD}}\right)-X_{H}\mathrm{VT}+\mathrm{VT}\frac{b}{c}\left(1-T_{\mathrm{DH}}-T_{\mathrm{HD}}\right)+\frac{2}{c}\left(1+\frac{b}{2}\right)\left(T_{\mathrm{HD}}-T_{\mathrm{DH}}\right)\right]}{\frac{2}{c}\left(1+\frac{b}{2}-X_{H}^{2}\frac{c}{2}\right)}=0$$

The equilibrium states are: ${\hat{X_{H}}}_{1}=1$, fixation of microbe H; ${\hat{X_{H}}}_{2}=0$, fixation of microbe D; and ${\hat{X_{H}}}_{3}=\frac{\mathrm{VT}-\sqrt{{\left(\mathrm{VT}\right)}^{2}-4\cdot \left(1-\mathrm{VT}\right)\left(T_{\mathrm{DH}}-T_{\mathrm{HD}}\right)\cdot \left[\mathrm{VT}\frac{b}{c}\left(1-T_{\mathrm{DH}}-T_{\mathrm{HD}}\right)+\frac{2}{c}\left(1+\frac{b}{2}\right)\left(T_{\mathrm{HD}}-T_{\mathrm{DH}}\right)\right]}}{2\left(1-\mathrm{VT}\right)\left(T_{\mathrm{DH}}-T_{\mathrm{HD}}\right)}$, polymorphism of H and D. If horizontal transmission is equal ($T_{\mathrm{HD}}=T_{\mathrm{DH}}=T$), the polymorphic equilibrium gets the form of ${\hat{X_{H}}}_{3}=\frac{b}{c}\left(1-2T\right)$, as in the perfect vertical transmission case.

${\hat{X_{H}}}_{1}=1$ is stable when $\frac{T_{\mathrm{DH}}}{T_{\mathrm{HD}}}<1+\frac{\mathrm{VT}\left[b\left(1-2T_{\mathrm{HD}}\right)-c\right]}{2T_{\mathrm{HD}}\left[1+\frac{b}{2}\left(1+\mathrm{VT}\right)-\frac{c}{2}\left(1-\mathrm{VT}\right)\right]}$ $\left(\frac{b-c}{2b}>T\right)$; ${\hat{X_{H}}}_{1}=0$ is stable when $1+\frac{\mathrm{VT}b\left(1-2T_{\mathrm{HD}}\right)}{2T_{\mathrm{HD}}\left[1+\frac{b}{2}\left(1+\mathrm{VT}\right)\right]}<\frac{T_{\mathrm{DH}}}{T_{\mathrm{HD}}}$ (unstable); and the polymorphic state is stable when $1+\frac{\mathrm{VT}\left[b\left(1-2T_{\mathrm{HD}}\right)-c\right]}{2T_{\mathrm{HD}}\left[1+\frac{b}{2}\left(1+\mathrm{VT}\right)-\frac{c}{2}\left(1-\mathrm{VT}\right)\right]}<\frac{T_{\mathrm{DH}}}{T_{\mathrm{HD}}}<1+\frac{\mathrm{VT}b\left(1-2T_{\mathrm{HD}}\right)}{2T_{\mathrm{HD}}\left[1+\frac{b}{2}\left(1+\mathrm{VT}\right)\right]}$ ($\frac{b-c}{2b}<T<\frac{1}{2}$). The conditions for the case of $T_{\mathrm{HD}}=T_{\mathrm{DH}}=T$ are given in the parentheses (see Appendix S1: Note 1, 1.2, for stability analysis).

In general, the next generation frequencies are affected by two aspects of the hosts interactions: the benefits and the costs to the hosts following the interactions, as well as the asymmetric horizontal transmission of the microbes (see Equation (7)). As vertical transmission (VT) decreases, the fitness effects of the host interaction diminish, and asymmetric horizontal transmission of microbes within the parent population becomes more influential. As a result, the equilibrium states can be altered, as well as the convergence times (Figure 3a), depending on the directions and the magnitudes of the two aspects mentioned above.

In the special case of symmetric horizontal transmission ($T_{\mathrm{HD}}=T_{\mathrm{DH}}=T$), there is no change in the equilibria and the stability conditions, compared to the case of VT=1. Nevertheless, the rate of converging to equilibrium is affected by the rate of vertical transmission. For the same initial conditions and parameters of the model, $\Delta X_{H}$ (see Equation (8)) is VT times lower compared to the case of VT=1. Therefore, the convergence to a certain equilibrium state is the fastest when vertical transmission is perfect (Figure 3a). However, even a low vertical transmission probability is sufficient to allow the population reaching the same equilibria, as long as it is still significant compared to other forces that neglected in the model, that is, mutation, migration, etc.

### 4.4 Evolutionary Game Theory Parallel Analysis of the Pure Strategies mHD Game Model

The mHD game model can be analyzed with the approach of Evolutionary Game Theory. Here we show the analysis of the model when vertical transmission is perfect (VT=1). The payoff $E\left(i,j\right)$ is the change of the Darwinian fitness to an individual that adopts strategy i, following a contest with an opponent that adopts strategy j. Since the baseline fitness is assumed to be 1, using Equations (3.1) and (3.2), the payoffs are (Table 2):

(9) $$\;E\left(i,j\right)=W\left(i,j\right)-1$$

**TABLE 2:** mHD game payoff matrix.

	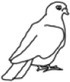	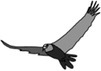
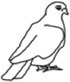	$\frac{b}{2}$	$\left(1+b\right)T_{\mathrm{DH}}-T_{\mathrm{HD}}$
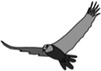	$b\left(1-T_{\mathrm{DH}}\right)+T_{\mathrm{HD}}-T_{\mathrm{DH}}$	$\frac{b-c}{2}$

*Note:* The payoff matrix of the mHD game model that is presented in the main text in Figures 1, 2, 3, and in Equation (9). The payoffs are shown for the case of perfect vertical transmission (VT=1).

The conditions for a strategy to be an ESS were given by Maynard Smith and Price (1973). In a population where individuals adopt one of the two strategies, i and j, strategy i is an ESS when: either

(10.1) $$\;E\left(i,i\right)>E\left(j,i\right)$$

or

(10.2) $$E\left(i,i\right)=E\left(j,i\right)\;\text{and}\;E\left(i,j\right)>E\left(j,j\right)$$

The strategy of microbe H satisfies Equations (10.1) and (10.2) and hence is an ESS when $\frac{2T_{\mathrm{HD}}+b-c}{2T_{\mathrm{HD}}\left(1+b\right)}\geq \frac{T_{\mathrm{DH}}}{T_{\mathrm{HD}}}$, and microbe D's strategy is an ESS when $\frac{2T_{\mathrm{HD}}+b}{2T_{\mathrm{HD}}\left(1+b\right)}\leq \frac{T_{\mathrm{DH}}}{T_{\mathrm{HD}}}$.

When $\frac{2T_{\mathrm{HD}}+b-c}{2T_{\mathrm{HD}}\left(1+b\right)}<\frac{T_{\mathrm{DH}}}{T_{\mathrm{HD}}}<\frac{2T_{\mathrm{HD}}+b}{2T_{\mathrm{HD}}\left(1+b\right)}$, there is no pure ESS strategy. In this case, however, there may be a mixed strategy that is an ESS. let I be a microbe that induces a mixed strategy of playing “Hawk” with probability $P_{I}$ (where $0<P_{I}<1$) and playing “Dove” otherwise. If I is an ESS, then (Bishop and Cannings 1978):

(11) $$\;E\left(H,I\right)=E\left(D,I\right)=E\left(I,I\right)$$

where:

$$E\left(m,I\right)=P_{m}\cdot \left[P_{I}\cdot E\left(H,H\right)+\left(1-P_{I}\right)\cdot E\left(H,D\right)\right]$$

$$+\left(1-P_{m}\right)\cdot \left[P_{I}\cdot E\left(D,H\right)+\left(1-P_{I}\right)\cdot E\left(D,D\right)\right]$$

and we define $P_{H}=1$, $P_{D}=0$. Solving Equation (11) for $P_{I}$ gives:

(12) $$\;P_{I}=\frac{b}{c}\left(1-2T_{\mathrm{DH}}\right)+\frac{2}{c}\left(T_{\mathrm{HD}}-T_{\mathrm{DH}}\right)$$

Strategy I satisfies Equations (10.1) and (10.2), and therefore mixed strategy I is an ESS when $\frac{2T_{\mathrm{HD}}+b-c}{2T_{\mathrm{HD}}\left(1+b\right)}<\frac{T_{\mathrm{DH}}}{T_{\mathrm{HD}}}<\frac{2T_{\mathrm{HD}}+b}{2T_{\mathrm{HD}}\left(1+b\right)}$.

If horizontal transmission is equal ($T_{\mathrm{HD}}=T_{\mathrm{DH}}=T$) and $T<\frac{1}{2}$, the strategy of microbe H satisfies Equations (10.1) and (10.2) and hence is an ESS when $\frac{b-c}{2b}\geq T$; microbe D's strategy is not an ESS; and a mixed strategy with probability of playing “Hawk” $P_{I}=\frac{b}{c}\left(1-2T\right)$, is an ESS when $\frac{b-c}{2b}<T<\frac{1}{2}$.

If there is no horizontal transmission (T=0), the classic genetic case results are obtained. A gene for Hawk‐behavior is an ESS when b≥c; a gene for Dove‐behavior can never be an ESS; and a mixed strategy with probability of playing “Hawk” $P_{I}=\frac{b}{c}$, is an ESS when b<c.

### 4.5 Description of the Mixed Strategies Model

In this section, we describe the mixed strategies model that is presented in Figure 4 in the main text. Here we consider the case where mixed strategies are allowed (Table 3). Let A and B, be microbes that induce strategies of playing “Hawk” with probability $P_{A}\left(H\right)$ and $P_{B}\left(H\right)$, respectively (and play “Dove” otherwise). We assume that a host of microbe B adopts the stable mixed strategy of the genes case (when b<c), thus $P_{B}\left(H\right)=b/c$. During interaction there is a probability T of microbe transmission from one host to the other. In addition, we assume that vertical transmission is perfect (VT=1).

**TABLE 3:** Parameters of the mixed strategies model.

Parameter	Description
$X_{A}$	Proportion of hosts carrying a standard microbiome plus microbes of type A
$X_{B}$	Proportion of hosts carrying a standard microbiome plus microbes of type B
b	Fitness benefit to the host following obtaining a resource
c	Fitness cost to the host due to injury during an interaction
$P_{A}\left(H\right)$	The probability of a hosts carrying microbe A to play “Hawk” during an interaction, and $1-P_{A}\left(H\right)$ is the probability to play “Dove”
$P_{B}\left(H\right)=\frac{b}{c}$	The probability of a hosts carrying microbe B to play “Hawk” during an interaction, and $1-P_{B}\left(H\right)$ is the probability to play “Dove”
T	Transmission and establishment probability of a microbe during its host interaction with a host carrying a different microbe type

*Note:* The parameters of the mixed strategies model that is presented in the main text in Figure 4.

Using the above, the frequencies in the next generation are:

(13.1) $$\;X_{A}^{\prime }=\frac{X_{A}^{2}W\left(A,A\right)+X_{A}X_{B}W\left(A,B\right)\;}{\bar{\omega }}$$

(13.2) $$\;X_{B}^{\prime }=\frac{X_{B}^{2}W\left(B,B\right)+X_{B}X_{A}W\left(B,A\right)}{\bar{\omega }}$$

The fitness following an interaction is given by:

(13.3) $$\;W\left(M,m\right)=\sum_{i=H,D}p_{M_{i}}\sum_{j=H,D}p_{m_{j}}\left[\left(1-T\right)\left(1+E\left(i,j\right)\right)+T\left(1+E\left(j,i\right)\right)\right]$$

where M and m are the microbe types (M and m can each be A or B), $p_{M_{i}}$ is the probability of a host to play strategy i while hosting microbe M, and $E\left(i,j\right)$ are the HD game payoffs (Figure 1).

The mean fitness of the population in each generation is as follows:

(13.4) $$\;\bar{\omega }=X_{A}^{2}W\left(A,A\right)+X_{A}X_{B}\left(W\left(A,B\right)+W\left(B,A\right)\right)+X_{B}^{2}W\left(B,B\right)$$

### 4.6 Equilibria and Stability of the Mixed Strategies Model

Solving for $\Delta X_{A}=0$,

(14) $$\;\Delta X_{A}=X_{A}\;X_{B}\frac{X_{A}\left[W\left(A,A\right)-W\left(A,B\right)-W\left(B,A\right)+W\left(B,B\right)\right]+W\left(A,B\right)-W\left(B,B\right)}{\bar{\omega }}=0$$

gives the following three equilibrium states: ${\hat{X_{A}}}_{1}=0$, fixation of microbe B; ${\hat{X_{A}}}_{2}=1$, fixation of microbe A; and ${\hat{X_{A}}}_{3}=\frac{2T}{1-\frac{P_{A}\left(H\right)}{b/c}}$, polymorphism of A and B.

${\hat{X_{A}}}_{1}=0$ is stable when $\frac{b}{c}<P_{A}\left(H\right)$; ${\hat{X_{A}}}_{2}=1$ is stable when $\frac{b}{c}\left(1-2T\right)<P_{A}\left(H\right)<\frac{b}{c}$; and the polymorphic state is stable when $P_{A}\left(H\right)<\frac{b}{c}\left(1-2T\right)$ (see Appendix S1: Note 1, 1.3, for stability analysis).

## Author Contributions

**Yonatan Bendett:** data curation (lead), formal analysis (lead), investigation (equal), methodology (equal), software (lead), visualization (equal), writing – original draft (lead). **Lilach Hadany:** conceptualization (lead), data curation (supporting), formal analysis (supporting), funding acquisition (lead), investigation (equal), methodology (equal), project administration (lead), resources (lead), supervision (lead), validation (lead), visualization (equal), writing – review and editing (lead).

## Conflicts of Interest

The authors declare no conflicts of interest.

## Code Availability

The figures code is available at GitHub: https://github.com/ybendett/Little‐Peacemakers‐‐‐figures‐code.

## Supporting information


Appendix S1.

## Data Availability

The authors have nothing to report.
