# Supplementary material for: Little Peacemakers: Microbes Can Promote Nonviolent Conflict Resolution by Their Hosts
Source: Ecol Evol. 2025 Apr 16;15(4):e71129. doi: 10.1002/ece3.71129 (PMC12000772; doi:10.1002/ece3.71129)
Supplement: Supplementary file 1 — Appendix S1. [file ECE3-15-e71129-s001.docx]

**Supplementary Information**

**Supplementary Note 1**

**1.1 Pure strategies mHD game model- stability analysis**

In the Methods section, we find the equilibrium states of the pure strategies mHD game model: ${\hat{X_{H}}}_{1}=1$, fixation of microbe $H$; ${\hat{X_{H}}}_{2}=0$, fixation of microbe $D$; and ${\hat{X_{H}}}_{3}=\frac{b}{c}\left( 1-2T_{DH} \right)+\frac{2}{c}(T_{HD}-T_{DH})$, polymorphism of $H$ and $D$.

An equilibrium state $\hat{X_{H}}$ is stable when:

$$\left( S.1 \right) \left| \left( \frac{\partial X_{H}^{'}}{\partial X_{H}} \right)_{X_{H}=\hat{X_{H}}} \right|<1$$

Using equations $(2)$-$(5)$, in the case of perfect vertical transmission ($VT=1$), we define:

$\left( S.2.1 \right) \lambda_{1}\equiv\left( \frac{\partial X_{H}^{'}}{\partial X_{H}} \right)_{X_{H}={\hat{X_{H}}}_{1}}=\frac{W\left( D,H \right)}{W\left( H,H \right)}=\frac{1+\left( 1+b \right)T_{DH}-T_{HD}}{1+\frac{b-c}{2}}$

$\left( S.2.2 \right) \lambda_{2}\equiv\left( \frac{\partial X_{H}^{'}}{\partial X_{H}} \right)_{X_{H}={\hat{X_{H}}}_{2}}=\frac{W\left( H,D \right)}{W\left( D,D \right)}=\frac{1+b-T_{DH}\left( 1+b \right)+T_{HD}}{1+\frac{b}{2}}$

$\left( S.2.3 \right) \lambda_{3}\equiv\left( \frac{\partial X_{H}^{'}}{\partial X_{H}} \right)_{X_{H}={\hat{X_{H}}}_{3}}=\frac{W\left( H,H \right)W\left( H,D \right)-2W\left( H,H \right)W\left( D,D \right)+W\left( D,H \right)W\left( D,D \right)}{W\left( H,D \right)W\left( D,H \right)-W\left( H,H \right)W\left( D,D \right)}=$

$=\frac{\frac{c}{2}\left[ 1+T_{DH}\left( 1+b \right)-T_{HD} \right]}{\left[ 1+\left( 1+b \right)T_{DH}-T_{HD} \right]\cdot\left[ 1+b-T_{DH}\left( 1+b \right)+T_{HD} \right]-\left( 1+\frac{b-c}{2} \right)\cdot\left( 1+\frac{b}{2} \right)}$

When $\left| \lambda_{i} \right|<1$ the equilibrium state ${\hat{X_{H}}}_{i}$ is stable (equation $\left( S.1 \right)$): ${\hat{X_{H}}}_{1}=1$ is stable when $\frac{2T_{HD}+b-c}{2T_{HD}\left( 1+b \right)}>\frac{T_{DH}}{T_{HD}}$; ${\hat{X_{H}}}_{1}=0$ is stable when $\frac{2T_{HD}+b}{2T_{HD}\left( 1+b \right)}<\frac{T_{DH}}{T_{HD}}$; and ${\hat{X_{H}}}_{3}=\frac{b}{c}\left( 1-2T_{DH} \right)+\frac{2}{c}(T_{HD}-T_{DH} )$is stable when $\frac{2T_{HD}+b-c}{2T_{HD}\left( 1+b \right)}<\frac{T_{DH}}{T_{HD}}<\frac{2T_{HD}+b}{2T_{HD}\left( 1+b \right)}$.

**1.2 Pure strategies mHD game with imperfect vertical transmission- stability analysis**

In the Methods section, we find the equilibrium states of the pure strategies mHD game model with imperfect vertical transmission: ${\hat{X_{H}}}_{1}=1$, fixation of microbe $H$; ${\hat{X_{H}}}_{2}=0$, fixation of microbe $D$; and ${\hat{X_{H}}}_{3}=\frac{VT-\sqrt{\left( VT \right)^{2}-4\cdot\left( 1-VT \right)\left( T_{DH}-T_{HD} \right)\cdot\left[ VT\frac{b}{c}\left( 1-T_{DH}-T_{HD} \right)+\frac{2}{c}\left( 1+\frac{b}{2} \right)\left( T_{HD}-T_{DH} \right) \right]}}{2\left( 1-VT \right)\left( T_{DH}-T_{HD} \right)}$, polymorphism of $H$ and $D$. Using equations $(7)$,$(8)$, in the case of $VT\neq1$, we define:

$\left( S.3.1 \right) \lambda_{1}\equiv\left( \frac{\partial X_{H}^{'}}{\partial X_{H}} \right)_{X_{H}={\hat{X_{H}}}_{1}}=\frac{VT\left[ 1+T_{DH}\left( 1+b \right)-T_{HD} \right]+\left( 1-VT \right)\left( 1-T_{HD}+T_{DH} \right)\left( 1+\frac{b}{2}-\frac{c}{2} \right)}{1+\frac{b}{2}-\frac{c}{2}}$

$\left( S.3.2 \right) \lambda_{2}\equiv\left( \frac{\partial X_{H}^{'}}{\partial X_{H}} \right)_{X_{H}={\hat{X_{H}}}_{2}}=\frac{VT\left( 1+b-T_{DH}\left( 1+b \right)+T_{HD} \right)+\left( 1-VT \right)\left( 1+T_{HD}-T_{DH} \right)\left( 1+\frac{b}{2} \right)}{1+\frac{b}{2}}$

$\left( S.3.3 \right) \lambda_{3}\equiv\left( \frac{\partial X_{H}^{'}}{\partial X_{H}} \right)_{X_{H}={\hat{X_{H}}}_{3}}=VT\frac{\left( 1+\frac{b}{2} \right)\left[ \left( 1+b \right)\left( 1-T_{DH} \right)+T_{HD} \right]}{\left( 1+\frac{b}{2}-\frac{c}{2}X_{H} \right)^{2}}+$

$+VT\frac{{\hat{X_{H}}}_{3}\left( 1+\frac{b}{2} \right)\left[ 2\left( T_{DH}-T_{HD} \right)-b\left( 1-2T_{DH} \right)-c \right]}{\left( 1+\frac{b}{2}-\frac{c}{2}X_{H} \right)^{2}}+VT\frac{{\hat{X_{H}}}_{3}^{2} \frac{c}{2}\left[ \left( 1+b \right)\left( 1-T_{DH} \right)+T_{DH} \right]}{\left( 1+\frac{b}{2}-\frac{c}{2}X_{H} \right)^{2}}+$

$+\left( 1-VT \right)\left[ 1+T_{HD}-T_{DH}+2{\hat{X_{H}}}_{3}\left( T_{DH}-T_{HD} \right) \right]$

When $\left| \lambda_{i} \right|<1$ the equilibrium state ${\hat{X_{H}}}_{i}$ is stable (equation $\left( S.1 \right)$): ${\hat{X_{H}}}_{1}=1$ is stable when $\frac{T_{DH}}{T_{HD}}<1+\frac{VT\left[ b\left( 1-2T_{HD} \right)-c \right]}{2T_{HD}\left[ 1+\frac{b}{2}\left( 1+VT \right)-\frac{c}{2}\left( 1-VT \right) \right]}$; ${\hat{X_{H}}}_{1}=0$ is stable when $1+\frac{VTb\left( 1-2T_{HD} \right)}{2T_{HD}\left[ 1+\frac{b}{2}\left( 1+VT \right) \right]}<\frac{T_{DH}}{T_{HD}}$; and the polymorphic state is stable when $1+\frac{VT\left[ b\left( 1-2T_{HD} \right)-c \right]}{2T_{HD}\left[ 1+\frac{b}{2}\left( 1+VT \right)-\frac{c}{2}\left( 1-VT \right) \right]}<\frac{T_{DH}}{T_{HD}}<1+\frac{VTb\left( 1-2T_{HD} \right)}{2T_{HD}\left[ 1+\frac{b}{2}\left( 1+VT \right) \right]}$.

**1.3 Mixed strategies model- stability analysis**

In the Methods section, we find the equilibrium states of the mixed strategies mHD game model with imperfect vertical transmission: ${\hat{X_{A}}}_{1}=0$, fixation of microbe $B$; ${\hat{X_{A}}}_{2}=1$, fixation of microbe $A$; and ${\hat{X_{A}}}_{3}=\frac{2T}{1-\frac{P_{A}}{b/c}}$, polymorphism of $A$ and $B$. Using equation $(13)$,$(14)$, we define:

$\left( S.4.1 \right) \lambda_{1}\equiv\left( \frac{\partial X_{A}^{'}}{\partial X_{A}} \right)_{X_{A}={\hat{X_{A}}}_{1}}=\frac{W\left( A,B \right)}{W\left( B,B \right)}=1+\frac{bt\left( \frac{b}{c}-P_{A} \right)}{1+\frac{b}{2}\left( 1-\frac{b}{c} \right)}$

$\left( S.4.2 \right) \lambda_{2}\equiv\left( \frac{\partial X_{A}^{'}}{\partial X_{A}} \right)_{X_{A}={\hat{X_{A}}}_{2}}=\frac{W\left( B,A \right)}{W\left( A,A \right)}=1+\frac{P_{A}^{2}\frac{c}{2}-P_{A}b\left( 1-T \right)+\frac{b^{2}}{2c}\left( 1-2T \right)}{1+\frac{b}{2}-\frac{cP_{A}^{2}}{2}}$

$\left( S.4.3 \right) \lambda_{3}\equiv\left( \frac{\partial X_{A}^{'}}{\partial X_{A}} \right)_{X_{A}={\hat{X_{A}}}_{3}}=\frac{W\left( A,A \right)W\left( A,B \right)-2W\left( A,A \right)W\left( B,B \right)+W\left( B,A \right)W\left( B,B \right)}{W\left( A,B \right)W\left( B,A \right)-W\left( A,A \right)W\left( B,B \right)}=$

$=1+\frac{bT\left[ P_{A}-\frac{b}{c}\left( 1-2T \right) \right]}{1+\frac{{2b}^{2}T}{c}\left( 1-T \right)+\frac{b}{2}\left( 1-\frac{b}{c} \right)}$

When $\left| \lambda_{i} \right|<1$ the equilibrium state ${\hat{X_{A}}}_{i}$ is stable (equation $\left( S.1 \right)$): ${\hat{X_{A}}}_{1}=0$ is stable when $\frac{b}{c}<P_{A}$ ; ${\hat{X_{A}}}_{2}=1$ is stable when $\frac{b}{c}\left( 1-2T \right)<P_{A}<\frac{b}{c}$ ; and ${\hat{X_{A}}}_{3}=\frac{2T}{1-\frac{P_{A}}{b/c}}$ is stable when $P_{A}<\frac{b}{c}\left( 1-2T \right)$.

**Supplementary Note 2**

**Pure strategies mHD game model- the effect of the parameter** $\boldsymbol{c}$

In the main text in Fig 2, we show the effect of horizontal transmission rates on the stable equilibria of the mHD game model. In Fig 2. we set $c$ to be constant, $c=0.1$. Here we demonstrate the effect of altering the value of this parameter. The parameter $c$, the fitness cost due to injury, only affects the outcomes of Hawk-Hawk interactions. When all other parameters are the same, as $c$ increases, the cost of injury is higher and ‘Hawk’ strategy is less successful; and vice versa when $c$ decreases. Compare Fig. 2 ($c=0.1$) in the main text to Supplementary Fig. S1 ($c=0.2$) and Supplementary Fig. S2 ($c=0.05$).


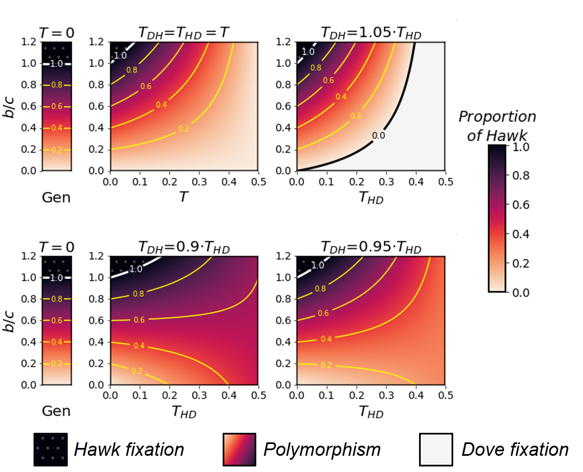


**Supplementary Fig. S1 | Horizontal transmission rates affect the stable equilibria of the mHD game (**$\boldsymbol{c=0.2}$**).**

The expected equilibrium proportion of hosts carrying $H$ microbe in population, for different $b/c$ ratio (y-axis) and different values of horizontal transmission probability (x-axis), plotted for (a) $T_{DH}/T_{HD}=1$, (b) $T_{DH}/T_{HD}=1.05$, (c) $T_{DH}/T_{HD}=0.9$, and (d) $T_{DH}/T_{HD}=0.95$. $c=0.2$.

The black dotted area represents the range of parameters in which microbe $H$ takes over the population, the colored area represents a range of parameters in which microbe $H$ and $D$ microbe reach stable polymorphism, and the white area represents the range of parameters in which microbe $D$ takes over the population. For derivation see Methods.


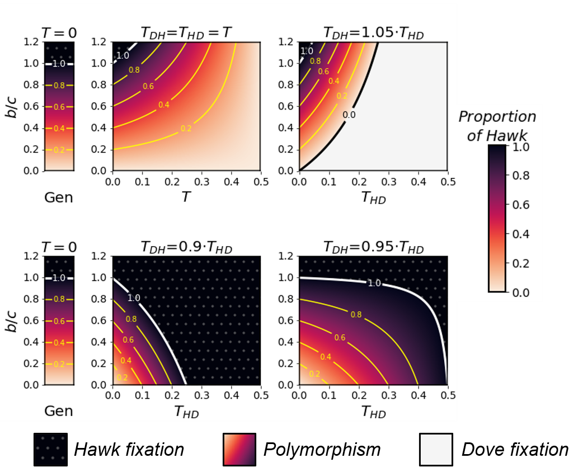


**Supplementary Fig. S2 | Horizontal transmission rates affect the stable equilibria of the mHD game (**$\boldsymbol{c=0.05}$**).**

The expected equilibrium proportion of hosts carrying $H$ microbe in population, for different $b/c$ ratio (y-axis) and different values of horizontal transmission probability (x-axis), plotted for (a) $T_{DH}/T_{HD}=1$, (b) $T_{DH}/T_{HD}=1.05$, (c) $T_{DH}/T_{HD}=0.9$, and (d) $T_{DH}/T_{HD}=0.95$. $c=0.05$.

The black dotted area represents the range of parameters in which microbe $H$ takes over the population, the colored area represents a range of parameters in which microbe $H$ and $D$ microbe reach stable polymorphism, and the white area represents the range of parameters in which microbe $D$ takes over the population. For derivation see Methods.

**Supplementary Note 3**

**Pure strategies mHD game model- Mean fitness**

In the main text in Fig. 2 and Fig. 3b, we show that whenever mHD leads to increased frequency of $D$ it further leads to increased mean fitness (Fig. 3b), compare to the genetic HD game for the same parameters. Here we show additional figures (Supplementary Figs. 3-5) that demonstrate the difference in mean fitness between the two cases, for broader range of parameters.


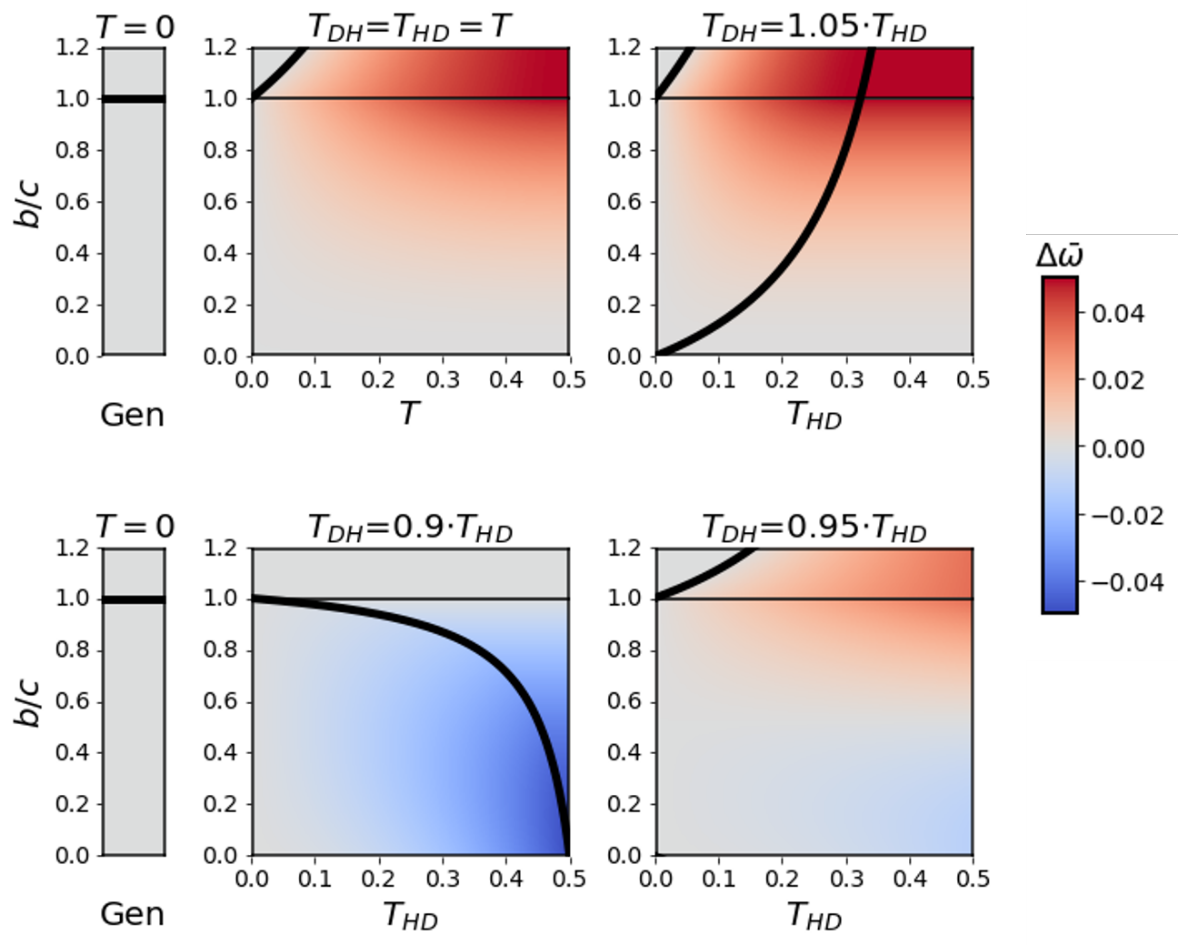


**Supplementary Fig. S3 | Mean fitness comparison between the mHD game and the classic HD game model (**$\boldsymbol{c=0.1}$**)**

The mean fitness differences between the mHD game and the genetic HD game evaluated in the stable equilibrium state ($\Delta\bar{\omega}=\bar{\omega}_{microbes HDG}-\bar{\omega}_{genetic HDG}$), for different $b/c$ ratio (y-axis) and different values of horizontal transmission probability (x-axis), plotted for (a) $T_{DH}/T_{HD}=1$, (b) $T_{DH}/T_{HD}=1.05$, (c) $T_{DH}/T_{HD}=0.9$, and (d) $T_{DH}/T_{HD}=0.95$. $c=0.1$.

The red area represents the range of parameters where the mean fitness of the population in the microbial case is higher than in the genetic case (for the same $b$ and $c$), and the blue area represents the range of parameters where the mean fitness in the genetic case is higher. The solid lines refer to the equilibrium states as shown in Fig 2 in the main text.


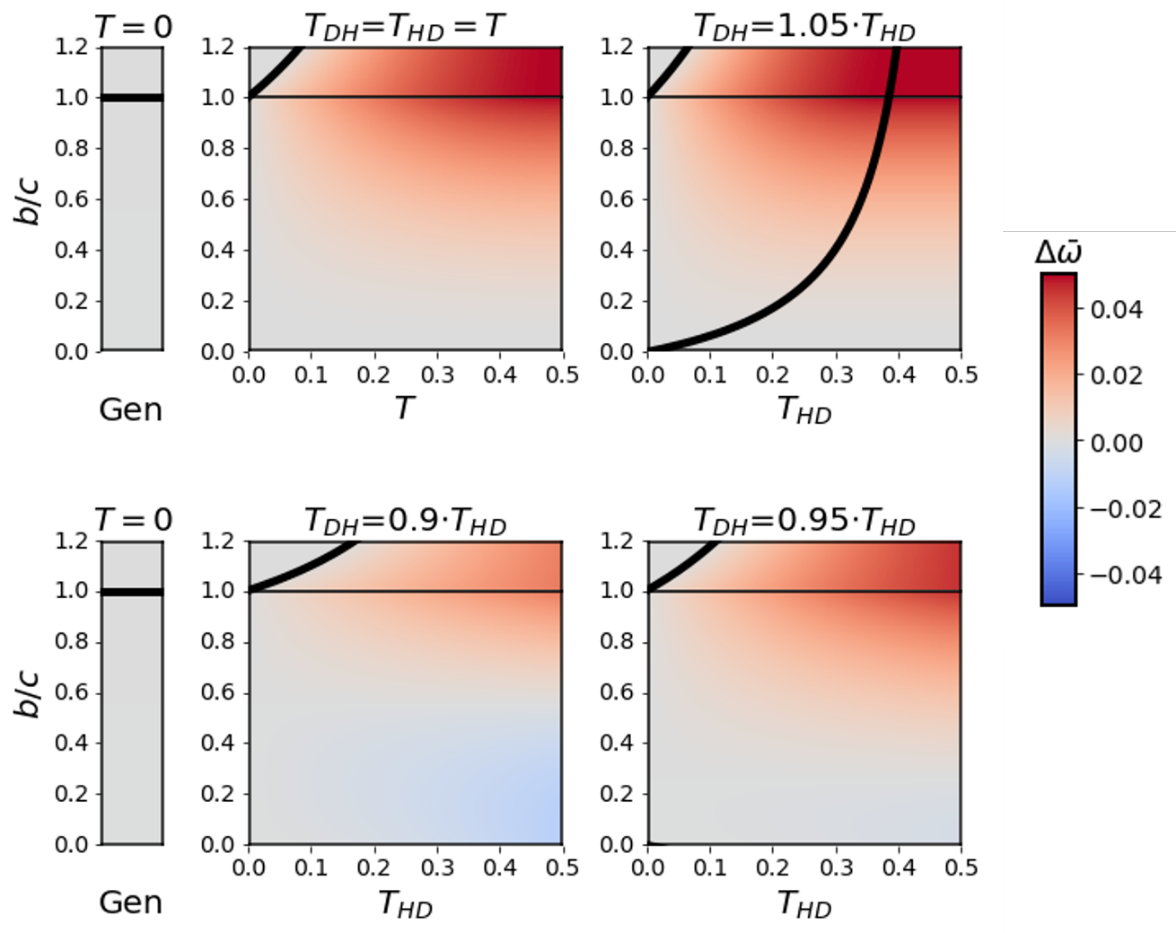


**Supplementary Fig. S4 | Mean fitness comparison between the mHD game and the classic HD game model (**$\boldsymbol{c=0.2}$**)**

The mean fitness differences between the mHD game and the genetic HD game evaluated in the stable equilibrium state ($\Delta\bar{\omega}=\bar{\omega}_{microbes HDG}-\bar{\omega}_{genetic HDG}$), for different $b/c$ ratio (y-axis) and different values of horizontal transmission probability (x-axis), plotted for (a) $T_{DH}/T_{HD}=1$, (b) $T_{DH}/T_{HD}=1.05$, (c) $T_{DH}/T_{HD}=0.9$, and (d) $T_{DH}/T_{HD}=0.95$. $c=0.2$.

The red area represents the range of parameters where the mean fitness of the population in the microbial case is higher than in the genetic case (for the same $b$ and $c$), and the blue area represents the range of parameters where the mean fitness in the genetic case is higher. The solid lines refer to the equilibrium states as shown in Supplementary Fig. S1.


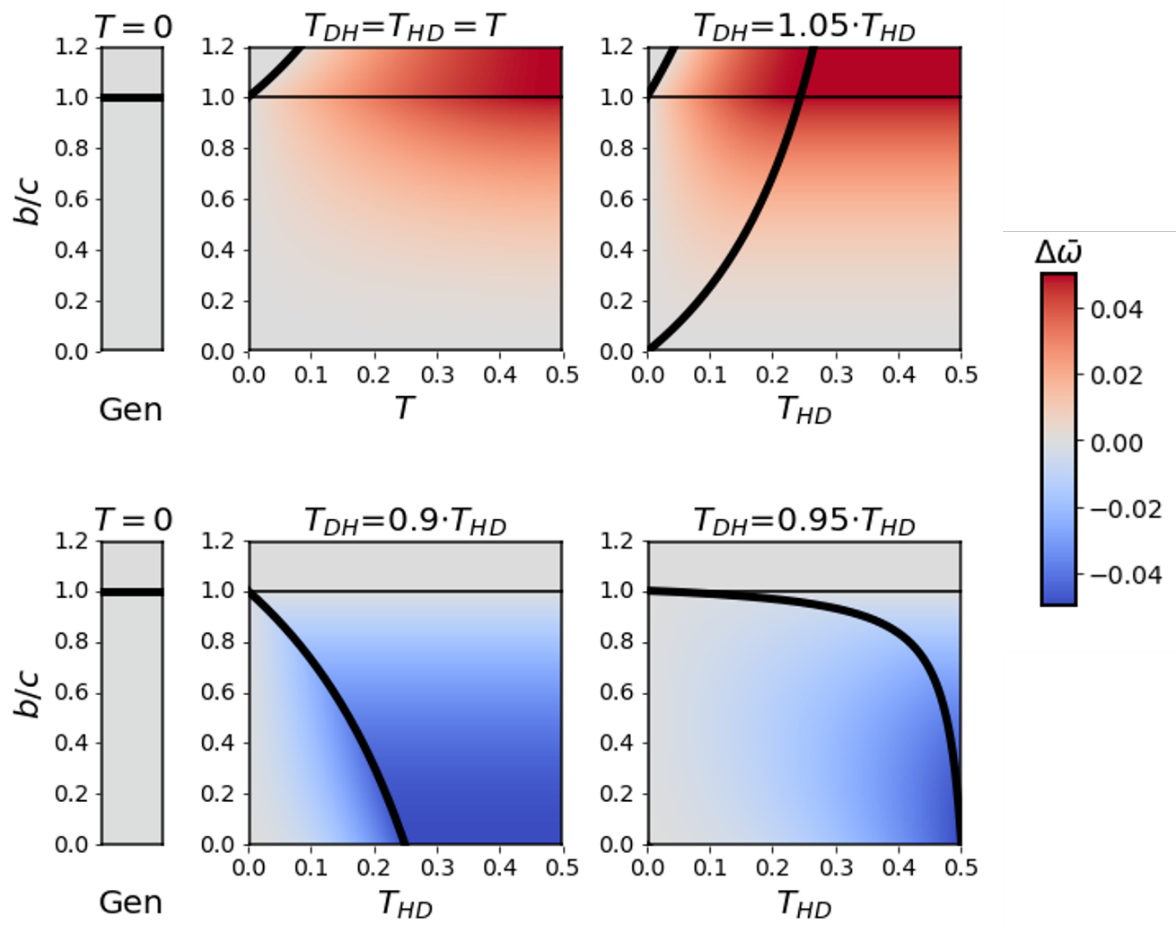


**Supplementary Fig. S5 | Mean fitness comparison between the mHD game and the classic HD game model (**$\boldsymbol{c=0.05}$**)**

The mean fitness differences between the mHD game and the genetic HD game evaluated in the stable equilibrium state ($\Delta\bar{\omega}=\bar{\omega}_{microbes HDG}-\bar{\omega}_{genetic HDG}$), for different $b/c$ ratio (y-axis) and different values of horizontal transmission probability (x-axis), plotted for (a) $T_{DH}/T_{HD}=1$, (b) $T_{DH}/T_{HD}=1.05$, (c) $T_{DH}/T_{HD}=0.9$, and (d) $T_{DH}/T_{HD}=0.95$. $c=0.05$.

The red area represents the range of parameters where the mean fitness of the population in the microbial case is higher than in the genetic case (for the same $b$ and $c$), and the blue area represents the range of parameters where the mean fitness in the genetic case is higher. The solid lines refer to the equilibrium states as shown in Supplementary Fig. S2.

**Supplementary Note 4**

**Alternative representation of the pure strategies model.**

In this section we describe am alternative mathematical description of the model that is presented in Fig. 1-2 in the main text. Here we assume, that each type of microbe has a different horizontal transmission probability ($T_{DH}, T_{HD}$), and that vertical transmission is perfect ($VT=1$).

We assume there are $N_{D}$ hosts that carry microbe $D$, and $N_{H}$ hosts that carry microbe $H$, and interactions occur randomly in pairs. The probability that a host that carries microbe $i$ interacts with a host that carries the same microbe is: $P_{ii}=\frac{N_{i}-1}{\sum_{k=D,N} N_{k} - 1}$, and the probability that a host that carries microbe $i$ interacts with a host that carries microbe $j\neq i$ is: $P_{ij}=\frac{N_{j}}{\sum_{k=D,N} N_{k} - 1}$. During host interaction, microbes can be transmitted between interacting hosts with probabilities $T_{HD}$ and $T_{DH}$. $T_{HD}$ represents the probability of microbes of type $H$ being transmitted to a host carrying microbe of type $D$, taking over its niche, and likewise for $T_{DH}$. When two hosts that carry the same microbe interact, exchanges of microbes result with no effect ($T_{DD}=T_{HH}=0$).

After interactions, the number of hosts that carry microbe $D$ and microbe $H$ are denoted by $\check{N_{D}}$ and $\check{N_{H}}$, respectively. Where:

$\left( S.5.1 \right) \check{N_{D}}=N_{D}P_{DD}+N_{D}P_{DH}\left( 1-T_{HD} \right)+N_{H}P_{HD}T_{DH}=$

$=N_{D}\frac{N_{D}-1}{N_{D}+N_{H} - 1}+N_{D}\frac{N_{H}}{N_{D}+N_{H} - 1}\left( 1-T_{HD} \right)+N_{H}\frac{N_{D}}{N_{D}+N_{H} - 1}T_{DH}$

$\left( S.5.2 \right) \check{N_{H}}=N_{D}P_{DH}T_{HD}+N_{H}P_{HH}+N_{H}P_{HD}\left( 1-T_{DH} \right)=$

$=N_{D}\frac{N_{H}}{N_{D}+N_{H} - 1}T_{HD}+N_{H}\frac{N_{H}-1}{N_{D}+N_{H} - 1}+N_{H}\frac{N_{D}}{N_{D}+N_{H} - 1}\left( 1-T_{DH} \right)$

A matrix representation is:

$$\left( S.5.3 \right) \left( \begin{matrix} \check{N_{D}} \\ \check{N_{H}} \end{matrix} \right)=T\left( \begin{aligned} N_{D} \\ N_{H} \end{aligned} \right)=\left( \begin{matrix} P_{DD}+P_{DH}\left( 1-T_{HD} \right) & P_{HD}T_{DH} \\ P_{DH}T_{HD} & P_{HH}+P_{HD}\left( 1-T_{DH} \right) \end{matrix} \right)\left( \begin{aligned} N_{D} \\ N_{H} \end{aligned} \right)=$$

$=\left( \begin{matrix} \frac{N_{D}-1}{N_{D}+N_{H} - 1}+\frac{N_{H}}{N_{D}+N_{H} - 1}\left( 1-T_{HD} \right) & \frac{N_{D}}{N_{D}+N_{H} - 1}T_{DH} \\ \frac{N_{H}}{N_{D}+N_{H} - 1}T_{HD} & \frac{N_{H}-1}{N_{D}+N_{H} - 1}+\frac{N_{D}}{N_{D}+N_{H} - 1}\left( 1-T_{DH} \right) \end{matrix} \right)\left( \begin{aligned} N_{D} \\ N_{H} \end{aligned} \right)$

The transition matrix $T$ represents the transmission probabilities following the interactions. $T_{ij}$ stands for the probability of host that carry microbe $j$ will carry microbe $i$ after an interaction. A diagram that illustrates the dynamics of transmission is shown in Supplementary Fig. S6.


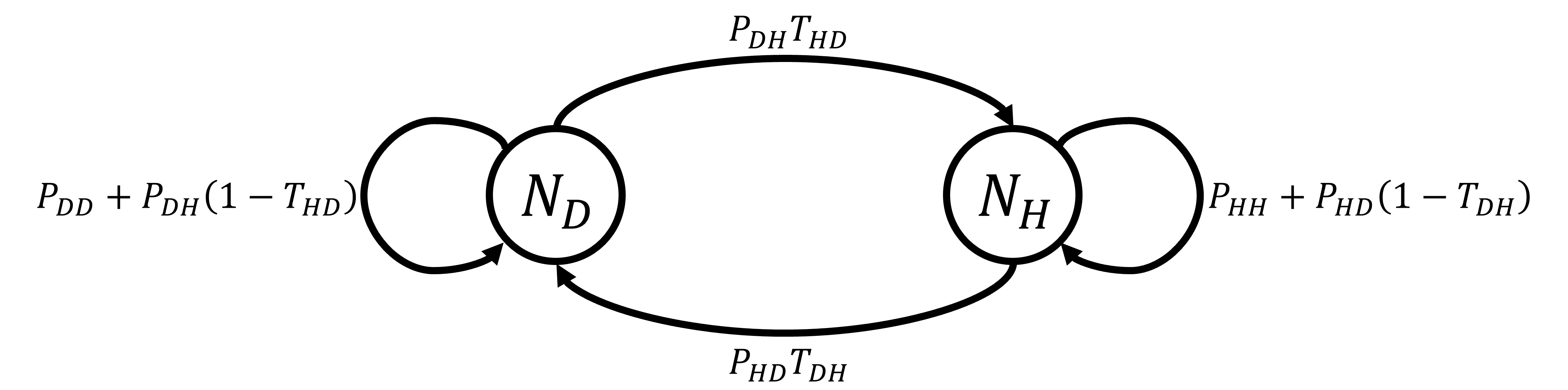


**Supplementary Fig. S6 | A diagram that illustrates the dynamics of transmission in a mHD game.**

A well-mixed population of $N_{D}$ and $N_{H}$ hosts that carry microbe $D$ and microbe $H$ respectively, interact randomly in pairs. The probability that a host that carries microbe $i$ interacts with a host that carries the same microbe is: $P_{ii}=\frac{N_{i}-1}{\sum_{k=D,N} N_{k} - 1}$, and the probability that a host that carries microbe $i$ interacts with a host that carries microbe $j\neq i$ is: $P_{ij}=\frac{N_{j}}{\sum_{k=D,N} N_{k} - 1}$. During host interaction, microbes can be transmitted between the interacting hosts. $T_{ij}$ represents the probability of microbes of type $i$ being transmitted to a host carrying microbe of type $j$, taking over its niche. When two interacting hosts carry the same microbe, there is no effect of microbial transmission ($T_{ii}=0$). The arrows represent the possible transitions and their probability. The corresponding matrix and mathematical equations are shown in equation $(S. 5)$.

The individuals interact with a Hawk-Dove (HD) game payoff (Fig 1), affecting fitness. We assume that the strategy a host adopts is determined by the microbe it carries before the interaction. Microbes of type $H$ induce their host to behave aggressively, i.e., behave always like a ‘Hawk’. Microbes of type $D$ induce their hosts to behave in a non-aggressive way, i.e., behave always like a ‘Dove’. After interactions occur, individuals reproduce according to their fitness. Microbes are transmitted vertically from parent to offspring, and offspring generation replaces the parent generation.

The fitness of the hosts depends on the interaction in which they were involved. Out of the $\check{N_{D}}$ individuals that carry microbe $D$ after the interaction, $\frac{N_{D}P_{DD}+N_{D}P_{DH}\left( 1-T_{HD} \right)}{\check{N_{D}}}$ of them carried microbe $D$ before the interaction, and $\frac{N_{H}X_{D}T_{DH}}{\check{N_{D}}}$ of them carried microbe $H$ before the interaction. $\frac{N_{D}P_{DD}}{\check{N_{D}}}$ were hosts of microbe $D$ that interacted with other hosts of microbe $D$, resulting in a fitness of $1+\frac{b}{2}$; $\frac{N_{D}P_{DH}\left( 1-T_{HD} \right)}{\check{N_{D}}}$ were hosts of microbe $D$ that interacted with hosts of microbe $H$, resulting in a fitness of $1$; and $\frac{N_{H}P_{HD}T_{DH}}{\check{N_{D}}}$ were hosts of microbe $H$ that interacted with hosts of microbe $D$, resulting in a fitness of $1+b$.

Out of the $\check{N_{H}}$ individuals that carry microbe $H$ after the interaction, $\frac{N_{H}P_{HH}+N_{H}P_{HD}\left( 1-T_{DH} \right)}{\check{N_{H}}}$ of them carried microbe $H$ before the interaction, and $\frac{N_{D}P_{DH}T_{HD}}{\check{N_{D}}}$ of them carried microbe $D$ before the interaction. $\frac{N_{H}P_{HH}}{\check{N_{H}}}$ were hosts of microbe $H$ that interacted with other hosts of microbe $h$, resulting in a fitness of $1+\frac{b-c}{2}$; $\frac{N_{H}P_{HD}\left( 1-T_{DH} \right)}{\check{N_{H}}}$ were hosts of microbe $H$ that interacted with hosts of microbe $D$, resulting in a fitness of $1+b$; and $\frac{N_{D}P_{DH}T_{HD}}{\check{N_{H}}}$ were hosts of microbe $D$ that interacted with hosts of microbe $H$, resulting in a fitness of $1$.

After reproduction and vertical transmission of the microbes, the number of newborn hosts that carry microbe $D$ and microbe $H$ are denoted by $N_{D}'$ and $N_{H}'$ , respectively. Where:

$\left( S.6.1 \right) N_{D}^{'}=\check{N_{D}}\left[ \frac{N_{D}P_{DD}}{\check{N_{D}}}\left( 1+\frac{b}{2} \right)+\frac{N_{D}P_{DH}\left( 1-T_{HD} \right)}{\check{N_{D}}}\left( 1 \right)+\frac{N_{H}P_{HD}T_{DH}}{\check{N_{D}}}\left( 1+b \right) \right]=$

$=N_{D}\frac{N_{D}-1}{N_{D}+N_{H} - 1}\left( 1+\frac{b}{2} \right)+N_{D}\frac{N_{H}}{N_{D}+N_{H} - 1}\left( 1-T_{HD} \right)+N_{H}\frac{N_{D}}{N_{D}+N_{H} - 1}T_{DH}\left( 1+b \right)$

$\left( S.6.2 \right) N_{H}^{'}=\check{N_{H}}\left[ \frac{N_{D}P_{DH}T_{HD}}{\check{N_{H}}}\left( 1 \right)+\frac{N_{H}P_{HD}\left( 1-T_{DH} \right)}{\check{N_{H}}}\left( 1+b \right)+\frac{N_{H}P_{HH}}{\check{N_{H}}}\left( 1+\frac{b-c}{2} \right) \right]=$

$=N_{D}\frac{N_{H}}{N_{D}+N_{H} - 1}T_{HD}+N_{H}\frac{N_{D}}{N_{D}+N_{H} - 1}\left( 1-T_{DH} \right)\left( 1+b \right)+N_{H}\frac{N_{H}-1}{N_{D}+N_{H} - 1}\left( 1+\frac{b-c}{2} \right)$

The equations that describe the frequencies of the microbes in the population are:

$\left( S.7.1 \right) X_{D}^{'}=\frac{N_{D}^{'}}{N_{D}^{'}+N_{H}^{'}}=$

$=\frac{X_{D}\left( \frac{N_{D}-1}{N_{T}} \right)\left( 1+\frac{b}{2} \right)+X_{D}X_{H}\left( 1-T_{HD}+\left( 1+b \right)T_{DH} \right)}{X_{D}\left( \frac{N_{D}-1}{N_{T}} \right)\left( 1+\frac{b}{2} \right)+X_{D}X_{H}\left( 1-T_{HD}+\left( 1+b \right)T_{DH} \right)+X_{H}X_{D}\left( \left( 1+b \right)\left( 1-T_{DH} \right)+ T_{HD} \right)+X_{H}\left( \frac{N_{H}-1}{N_{T}} \right)\left( 1+\frac{b-c}{2} \right)}$

$\left( S.7.2 \right) X_{H}^{'}=\frac{N_{H}^{'}}{N_{D}^{'}+N_{H}^{'}}=$

$=\frac{X_{H}X_{D}\left( \left( 1+b \right)\left( 1-T_{DH} \right)+ T_{HD} \right)+X_{H}\left( \frac{N_{H}-1}{N_{T}} \right)\left( 1+\frac{b-c}{2} \right)}{X_{D}\left( \frac{N_{D}-1}{N_{T}} \right)\left( 1+\frac{b}{2} \right)+X_{D}X_{H}\left( 1-T_{HD}+\left( 1+b \right)T_{DH} \right)+X_{H}X_{D}\left( \left( 1+b \right)\left( 1-T_{DH} \right)+ T_{HD} \right)+X_{H}\left( \frac{N_{H}-1}{N_{T}} \right)\left( 1+\frac{b-c}{2} \right)}$

Where $N_{T}=N_{D}+N_{H}$ is the number of individuals in the population, and $X_{i}=\frac{N_{i}}{N_{T}}$ is the frequency of microbe $i$. When the number of hosts that carry microbes $D$ and $H$ is large ($N_{D}\gg1$, $N_{H}\gg1$), then $\frac{N_{H}-1}{N_{T}}\approx\frac{N_{H}}{N_{T}}=X_{H}$, $\frac{N_{D}-1}{N_{T}}\approx\frac{N_{D}}{N_{T}}=X_{D}$, equations $\left( S.7 \right)$ get the form of:

$\left( S.8.1 \right) X_{D}^{'}=\frac{X_{D}^{2}\left( 1+\frac{b}{2} \right)+X_{D}X_{H}(1-T_{HD}+\left( 1+b \right)T_{DH})}{X_{D}^{2}\left( 1+\frac{b}{2} \right)+X_{D}X_{H}\left( 1-T_{HD}+\left( 1+b \right)T_{DH} \right)+X_{H}X_{D}\left( \left( 1+b \right)\left( 1-T_{DH} \right)+ T_{HD} \right)+X_{H}^{2}\left( 1+\frac{b-c}{2} \right)}$

$\left( S.8.2 \right) X_{H}^{'}=\frac{X_{H}^{2}\left( 1+\frac{b-c}{2} \right)+X_{H}X_{D}\left( \left( 1+b \right)\left( 1-T_{DH} \right)+ T_{HD} \right)}{X_{D}^{2}\left( 1+\frac{b}{2} \right)+X_{D}X_{H}\left( 1-T_{HD}+\left( 1+b \right)T_{DH} \right)+X_{H}X_{D}\left( \left( 1+b \right)\left( 1-T_{DH} \right)+ T_{HD} \right)+X_{H}^{2}\left( 1+\frac{b-c}{2} \right)}$

These are the same equations that are shown in equations $\left( 2 \right)-\left( 5 \right)$ in the Methods section, for infinitely large population. The equilibria and stability of the system are analyzed there.

**Constant population size and stochastic simulations.**

Now we assume the population is maintained in a constant size, due to resource limitation, territory size etc. After reproduction, the offspring population go through a bottleneck, maintaining a constant population size.

We model the bottleneck both deterministically and stochastically. In the deterministic model, the number of individuals decreases such that the frequencies of each microbe type in the population do not change. The frequencies in the next generation, $X_{D}^{'},X_{H}^{'}$, are given in equations $\left( S.7.1 \right)-\left( S.7.2 \right)$, and the numbers of offspring after the bottleneck are:

$$\left( S.9.1 \right) N_{D}^{'}=N_{T}\cdot X_{D}^{'}$$

$$\left( S.9.2 \right) N_{H}^{'}=N_{T}\cdot X_{H}^{'}$$

Where the population size is $N_{T}$.

We use Wright-Fisher model (Wright 1931; Fisher 1958) to model the bottleneck stochastically. The number of the offspring that carry microbe $i$ is binomially distributed, $Bin\left( N_{T},X_{i} \right)$, where $N_{T}$ is the size of the population after the bottleneck, and $X_{i}$ is the frequency of offspring that carry microbe $i$ before the bottleneck.

Examples of the population dynamics are shown in Supplementary Fig. S7-S8.


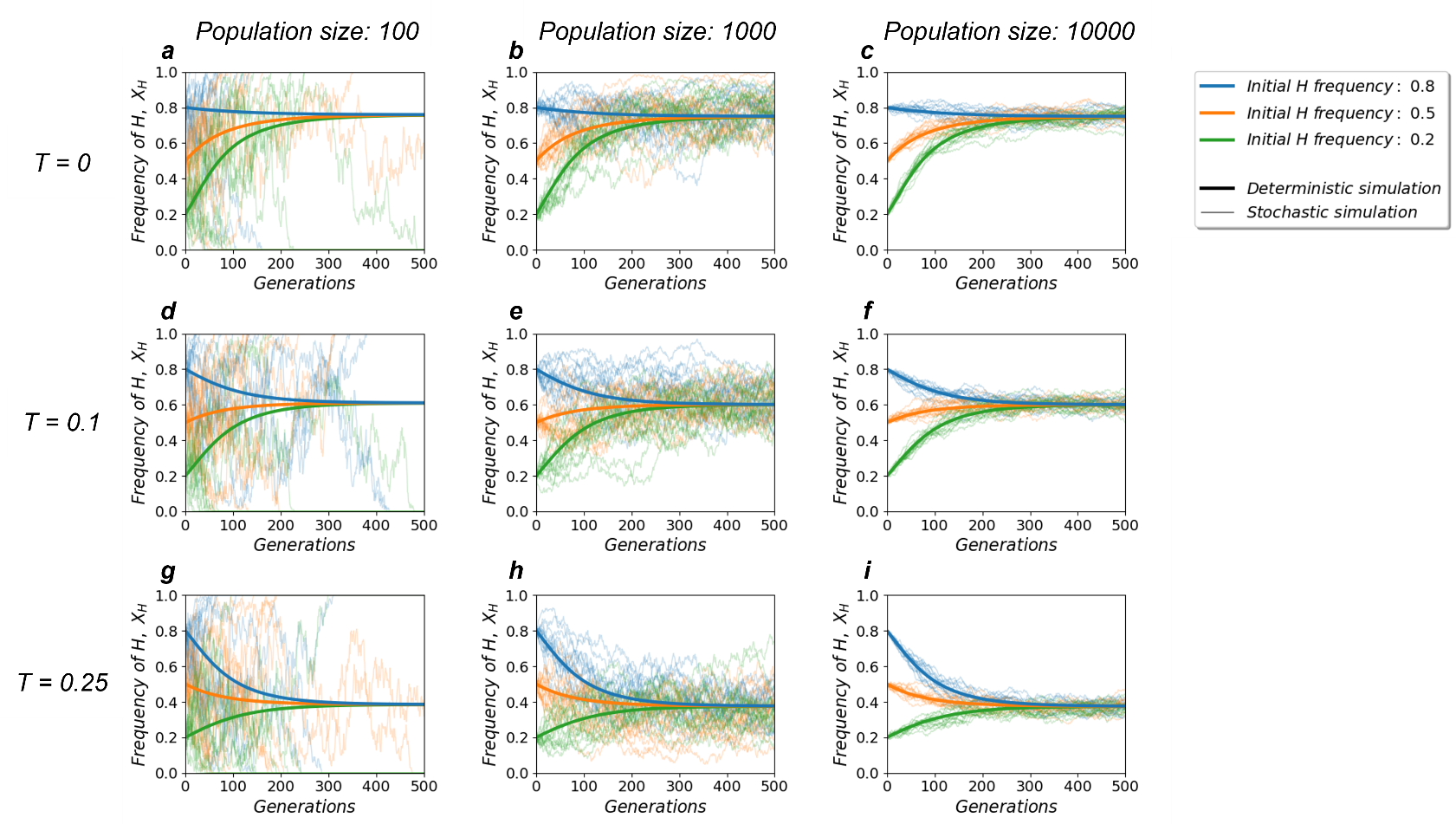


**Supplementary Fig. S7 | Deterministic and stochastic dynamics of mHD game model in constant size population.** $\boldsymbol{c=0.1,b=0.075}$**.**

Simulations of the population dynamics along 500 generations. Thick solid lines represent deterministic simulations of $H$ microbe frequency, where the colors refer to different initial frequencies: blue 0.8, orange 0.5, green 0.2. Thin lines represent individual stochastic simulations for the initial frequencies of $H$ microbe represented by the same color (15 simulations for each).

The dynamics are shown for symmetric horizontal transmission: **(a),(b),(c)** $T=0$; **(d),(e),(f)** $T=0.1$; **(g),(h),(i)** $T=0.25$. The frequency of hawk microbe (y-axis) is plotted as function of generations number (x-axis) for different constant size population: **(a),(d),(g)** 100 individuals, **(b),(e),(h)** 1000 individuals, **(c),(f),(i)** 10000 individuals.


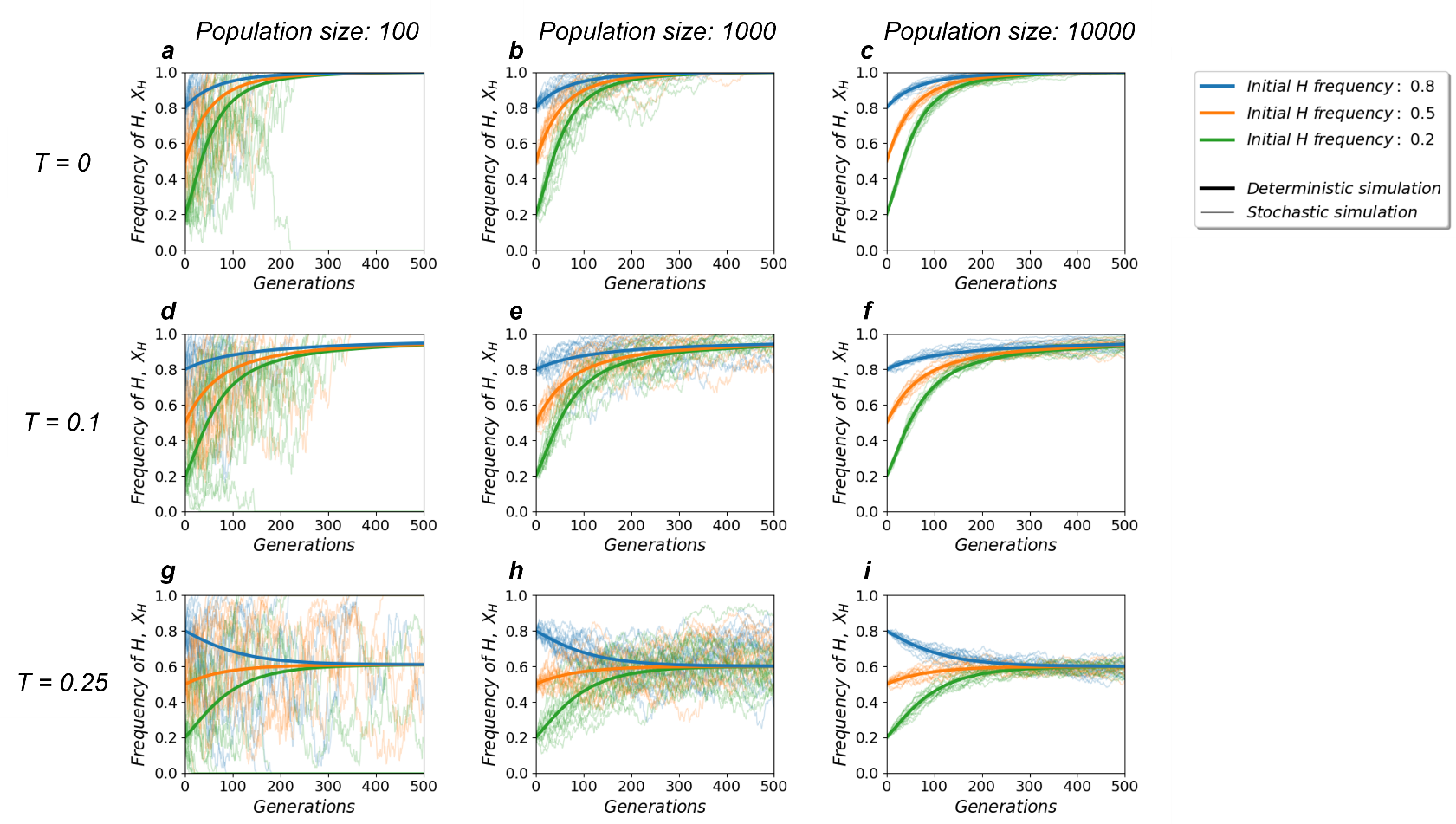


**Supplementary Fig. S8 | Deterministic and stochastic dynamics of mHD game model in constant size population.** $\boldsymbol{c=0.1,b=0.12}$**.**

Simulations of the population dynamics along 500 generations. Thick solid lines represent deterministic simulations of $H$ microbe frequency, where the colors refer to different initial frequencies: blue 0.8, orange 0.5, green 0.2. Thin lines represent individual stochastic simulations for the initial frequencies of $H$ microbe represented by the same color (15 simulations for each).

The dynamics are shown for symmetric horizontal transmission: **(a),(b),(c)** $T=0$; **(d),(e),(f)** $T=0.1$; **(g),(h),(i)** $T=0.25$. The frequency of hawk microbe (y-axis) is plotted as function of generations number (x-axis) for different constant size population: **(a),(d),(g)** 100 individuals, **(b),(e),(h)** 1000 individuals, **(c),(f),(i)** 10000 individuals.
